# A Triple-Site Gd_3_ Carborane Metal–Organic Framework toward Scalable Quantum Computing

**DOI:** 10.1021/acsami.5c06002

**Published:** 2025-06-12

**Authors:** Elena Bartolomé, Xiao-Bao Li, Ana Arauzo, Javier Luzón, Inés García-Rubio, José Giner Planas

**Affiliations:** † Consejo Superior de Investigaciones Científicas (CSIC), 54449 Institut de Ciència de Materials de Barcelona (ICMAB) , Campus UAB, Bellaterra, 08193 Barcelona, Spain; ‡ CSIC-Universidad de Zaragoza, and Departamento de Física de la Materia Condensada, Instituto de Nanociencia y Materiales de Aragón (INMA), 50009 Zaragoza, Spain; § Centro Universitario de Defensa (CUD), Carretera de Huesca s/n, 50090 Zaragoza, Spain

**Keywords:** lanthanide metal−organic framework, gadolinium, carborane-linker, molecular qubits, qudit, quantum computing

## Abstract

Metal–organic frameworks (MOFs) incorporating arrays of molecular spin qubits (quMOFs) offer a promising pathway toward scalable quantum computing. In this work, we introduce a novel quMOF, {[(Gd)_3_(*m*CB-L)_4_(NO_3_)­(DMF)_*x*_]_*n*_·Solv}, constructed with a carborane linker and Gd­(III) ions at three distinct coordination sites. We thoroughly characterize its magneto-thermal properties using dc/ac magnetometry, X-ray absorption spectroscopy, X-ray magnetic circular dichroism, and heat capacity measurements. The quantum computing potential is demonstrated through ab initio calculations and pulsed electron paramagnetic resonance on GdY-diluted analogues, revealing *T*_m_= 0.7 μs and Rabi oscillations persisting up to 50 K. Each of the three isolated Gd­(*i*) sites in GdY-MOFs functions as an 8-level qudit, accessible via X-band transitions. Notably, the triple-site Gd_3_ quMOF provides an unprecedented qudit with *d* = (2*S* + 1)^3^ = 512 states, capable of encoding up to 9 qubits, marking a significant advance in the scalability of molecular-based quantum computing systems.

## Graphical abstract

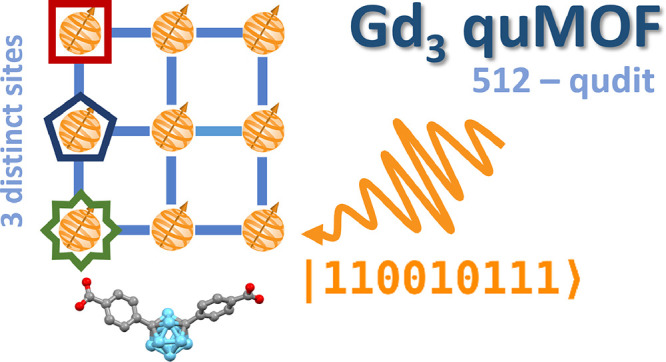

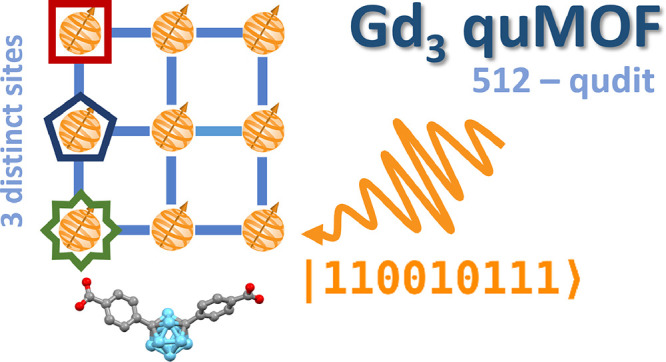

## Introduction

Quantum Computing (QC) has emerged as a revolutionary quantum technology that promises to transform numerous fields by solving complex problems that surpass the capabilities of classical supercomputers, with foreseen application in artificial intelligence (AI), machine learning, optimization, data analysis, and beyond.^1,2^ The basic unit of QC is the qubit, which represents a coherent superposition of two states (“0” and “1”). Various qubit platforms have been proposed to date, including superconducting circuits,^3^ quantum dots,^4^ trapped ions,^5^ neutral atoms, diamond vacancies, photons,^6^ topological qubits and molecules, each offering distinct strengths and challenges.^7,8^

Among these, molecular spin qubits present interesting advantages.^9^ Molecules are inherently quantum systems with discrete energy levels that can be finely tuned through chemical design. The bottom-up synthetic approach enables the scalable and reproducible production of identical qubits, pushing miniaturization to its limits. Furthermore, molecular qubits can be functionalized, deposited on diverse surfaces, or assembled into periodic arrays, providing exceptional flexibility for integration into quantum devices. The electron and nuclear spin state of these systems can be manipulated using microwave (MW) or radio frequency pulses, respectively, facilitating the operation of quantum gates (“qugates”) and algorithm execution. Advances in decoherence mitigation have led to molecules exhibiting coherence times as long as *T*_2_ ∼ 1 ms at 10 K, as demonstrated in the vanadium monomer ((d_20_-Ph_4_P)_2_[V­(C_8_S_8_)_3_]).^10^ Since 2016, interest has increasingly shifted toward lanthanide-based qubits, where tailored crystal fields enable the design of the desired quantum states. Molecules like bis­(phthalocyanine)­terbium­(III) (TbPc_2_)^11^ have already demonstrated compliance with DiVincenzo’s criteria for qubits,^12^ showing well-defined energy levels, scalability, long coherence times, efficient initialization, manipulation, readout, and capability for universal gate implementation. Recent progress has even enabled quantum error correction (QEC) within single lanthanide molecules,^13^ further underscoring their promise for quantum information processing.

Current research focuses on overcoming key challenges for the practical implementation of molecular qubits in QC,^8,14^ such as integrating and coupling molecules into hybrid solid-state devices,^9,15–17^ developing optically addressable molecular spins,^18,19^ and expanding the dimension of the accessible Hilbert space.^20^ Scaling up the number of states is essential to boost computational power and enable advanced quantum algorithms, thereby unlocking the true potential of QC.

Scaling can be achieved through two fundamentally distinct approaches. The first involves combining multiple two-level qubits within a molecular system. This can be achieved by integrating several qubits within a single molecule, as demonstrated with heterometallic lanthanide-based [LnLn’] ^21,22^ and [LnLn’Ln] ^13,23^ qugates, or by connecting molecular qubits through supramolecular chemistry, such as in Cr_7_Ni systems.^24^ The second approach leverages the internal states of magnetic molecules to access a greater number of quantum transitions within a *d*-level quantum system, known as a “qudit”. A single *d*-level qudit can efficiently encode *N* two-level qubits (*d* = 2^*N*^). Using qudits instead of conventional two-level qubits allows for more compact quantum architectures, the implementation of simplified or improved quantum algorithms^25,26^ and facilitates QEC protocols.^13,27,28^ Thus, qudits can significantly boost quantum logic in applications ranging from quantum computing,^29^ quantum sensing,^30^ and quantum simulation.^31,32^

Molecular 4-level qudits have been successfully realized in TbPc_2_ using the *I* = 3/2 nuclear spin of Tb­(III),^33^ which enabled the first demonstration of Grover’s algorithm,^11,34^ while the coupling of two Tb-qudits allowed creating a 16-level system in Tb_2_Pc_3_.^35^ Gd­(III) ion with a large spin *S* = 7/2 and no orbital contribution (*L* = 0), offers the possibility of producing qudits of larger dimensionality. The sensitivity of Gadolinium magnetic anisotropy to the local coordination gives rise to zero-field splittings, which can be used to shape qudits.^36–39^ Following this idea, a single-ion [GdW_30_] *d* = 8 qudit (equivalent to *N* = 3 qubits) was reported,^40^ and more recently, the synthesis of a dissymmetric [GdGd’] molecule allowed to build up a *d* = 64 qudit, hosting up to *N* = 6 addressable spin qubits.^41^ This is the highest number of qubits achieved in molecular QC so far.

Interestingly, Metal–Organic Frameworks (MOFs), composed of organic linkers and metal ions arranged in crystalline porous structures, provide ideal platforms to organize qubits into ordered arrays. These so-called “quMOFs” are promising for QC, enabling scalable and modular qubit structures, while allowing precise control over qubit spacing and magnetic dilution to reduce decoherence. Their porosity also supports quantum sensing^42–47^ and enables customization through guest inclusion.^48^ Qubits have been integrated into MOFs as paramagnetic metal centers in the nodes (e.g., Cu^2+^ for sensing^49^), metal centers within the building ligands,^50–52^ or as organic radicals.^42–47^

In particular, MOFs coordinating lanthanide (Ln) ions stand out for their unique magnetic, electronic and optical properties, making them promising materials for QC as well as for applications in magnetic refrigeration, luminescence, or catalysis. However, so far, only three Ln­(III) quMOFs have been reported for QC: (i) the Gd­(III)-based 3D MOF, [Gd­(bipyNO)_4_]­(TfO)_3_·*x*MeOH, where pulsed electron paramagnetic resonance (EPR) experiments on a diluted analogue revealed a spin–spin relaxation time *T*_m_ = 612 ns and a spin–lattice relaxation time *T*_1_ = 66 μs at 3.5 K, along with Rabi oscillations up to 40 K;^53^ (ii) the 2D Gd^III^Na^I^-based oxamato supramolecular coordination framework, with extended relaxation times (*T*_m_ = 4.25 μs at 8 K and *T*_1_ = 1.66 ms at 4 K) in a 0.12% magnetically diluted sample;^54^ and (iii) layered MOF [N­(C_2_H_5_)_4_]­[Ln_*x*_La_100–*x*_(CAN)_2_(H_2_O)], Ln = Gd­(III), Nd­(III), with *T*_m_ ≈ 2 μs at 3.2 K in 0.1–0.5% diluted samples.^55^ We note that Gd­(III)-based MOFs are also attractive candidates for low-temperature magnetic cooling,^16–18^ due to their large magnetocaloric effect (MCE), which is based on Gd spin-only *S* = 7/2 (maximum entropy can be obtained from *S*_max_ = Rln­(2*S* + 1)). The MCE performance of 4f compounds of various dimensions, including Gd 3D MOFs, has been recently reviewed.^19^

Recently, some of us reported the one-pot synthesis of a family of lanthanide MOFs using the carborane linker *m*CB-L = 1,7-di­(4-carboxyphenyl)-1,7-dicarba-*closo*-dodecarborane,^56^ with the general formula {[(Ln)_3_(*m*CB-L)_4_(NO_3_)­(DMF)_*x*_]_*n*_·Solv}. Icosahedral carboranes are a class of commercially available, stable boron-rich clusters^57–59^ known for their high hydrophobicity^60–66^ and unusual electronic structure, often regarded as inorganic 3D “aromatic” analogs of arenes.^57–59^ The steric bulkiness and acidity of the *m*CB-L linker facilitate the synthesis of multivariate MOFs incorporating any desired combination of lanthanides, enabling the exploration of complex magnetic materials and multifunctional materials with tailored properties.^67^ In previous studies, we highlighted the multifunctional properties of Tb, Dy MOFs,^68^ mixed Tb/Eu MOFs with exceptional luminescence,^56^ “self-refrigerated” GdLn (Ln = Tb, Dy, Eu) MOFs,^69^ and the first MOF incorporating eight different-sized rare-earth ions.^67^ The MOF structures, which are all isostructural, are formed by the repetition of Secondary Building Units (SBUs) containing three ions in distinct coordination environments, while the bulky carborane ligands separate adjacent chains to create the 3D architecture.

Importantly, the ability to create multilanthanide MOFs with distinct coordination sites opens promising possibilities for scaling the Hilbert space for QC. Notably, a Gd_3_ quMOF with three distinct sites could yield an unparalleled qudit with *d* = (2*S* + 1)^3^ = 512 states, equivalent to 9 two-level qubits, providing an exceptional platform for scaling Quantum Computing systems.

In this work, we report the synthesis and characterization of a triple-site Gd­(III) carborane-based MOF, {[(Gd)_3_(*m*CB-L)_4_(NO_3_)­(DMF)_*x*_]_*n*_Solv}, along with two magnetically diluted analogs. We first investigate its magneto-thermal properties using dc/ac magnetometry, XAS-XMCD, and heat capacity measurements. Second, we assess the quantum computing potential of these multisite Gd quMOFs through ab initio calculations and pulsed EPR experiments, highlighting their capacity to expand the Hilbert space.

## Results and Discussion

### Synthesis and Structure

For this work, we synthesized the carborane-based Gd­(III) MOF (***m*** **CB-Gd**) and two magnetically diluted analogs, ***m*** **CB-Gd**_**0.08%**_ and ***m*** **CB-Gd**_**1.76%**_, containing 0.08% and 1.76% Gd in Yttrium, respectively, using a simple one-pot method previously applied to the preparation of other Ln-based MOFs in the same family.^67^ Powder X-ray diffraction (PXRD) data confirms that these materials are isostructural with previously reported *m*CB-Ln MOFs (Figure S1),^56,67–69^ although with clear preferred orientation effects resulting from the highly anisotropic crystalline materials. The metals’ content in the mixed MOFs was determined by Inductively Coupled Plasma–Mass Spectrometry (ICP-MS), as detailed in the Experimental Section.

Figure 1 shows a representation of the structure for ***m*** **CB-Gd**. The secondary building unit (SBU) of this MOF is formed by three nonequivalent Gd­(III) ions —denoted Gd(1), Gd(2), Gd(3) —linearly arranged along the *a*-axis and capped by bridging or chelate-bridging *m*CB-L, chelate NO_3_^–^ and DMF molecules. The coordination environment of Gd2 is composed of seven O atoms, while Gd1 and Gd3 are octa-coordinated by O atoms (Figure 1a).

**Figure 1:**
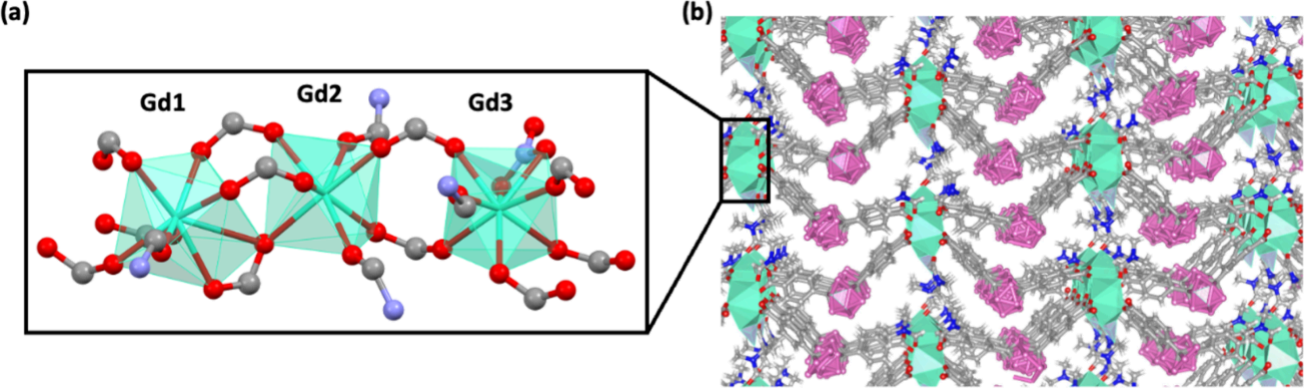
Representation of the crystal structure for ***m*** **CB-Gd**. (a) Detail of the Secondary Building Unit (SBU) containing three nonequivalent sites for Gd­(III): Gd(1), Gd(2), Gd(3). (b) View of the 3D structure showing the coordination of *m*CB-L to Gd atoms. Green polyhedra represents the Gd coordination spheres. H atoms are omitted for clarity. Color code: B, pink; C, gray; O, red; N, blue.

### Magnetic Properties

The static magnetic characterization of ***m*** **CB-Gd** is shown in Figure 2. The temperature-dependence of the χ*T* product at 300 K reaches a value of 7.81 emu K/mol, very close to the free-ion value of 7.88 emu K/mol expected for one Gd­(III) with *S* = 7/2 and *g* = 2.0 (Figure 2a). By decreasing the temperature, the χ*T* value remains nearly constant down to 50 K, and it only drops to a value of 7.46 emu K/mol at 1.8 K, as a result of the combined effect of very weak magnetic interactions between the Gd­(III) ions and single ion magnetic anisotropy (*vide infra* ab initio calculations). The linear fit of the 1/χ plot at high temperatures yields a Curie–Weiss temperature of θ = −2.84 K, pointing to the existence of overall antiferromagnetic (AF) interactions. In view of ab initio results, these AF interactions are mostly of dipolar origin.

**Figure 2:**
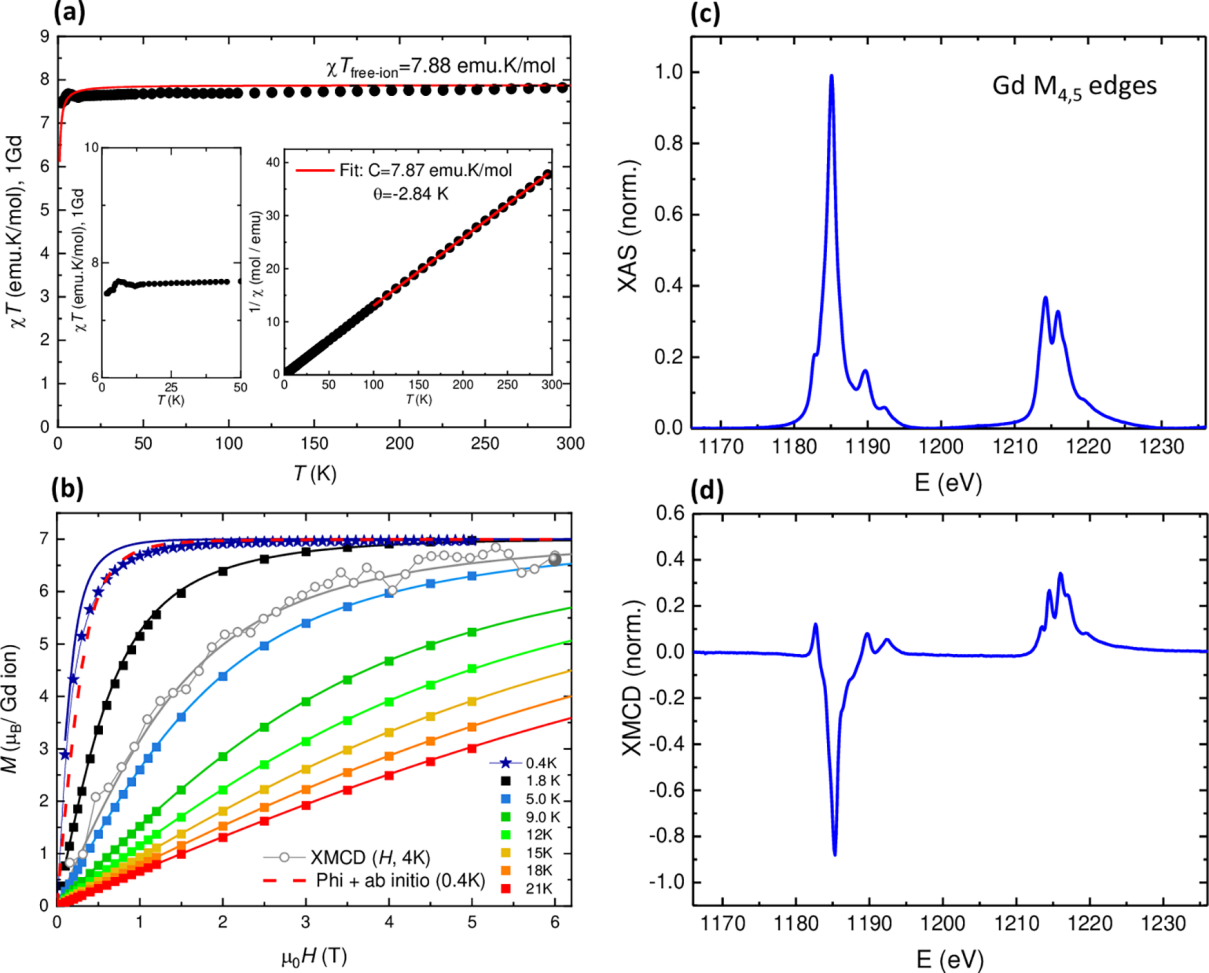
Dc magnetic properties of ***m*** **CB-Gd.** (a) χ*T* product as a function of the temperature at 0.1 T per Gd ion, and zoom of the low-*T* region. Red line: simulation using ab initio-calculated parameters. Right inset: 1/χ plot and Curie fit above 100 K. (b) Field-dependence of the magnetization, per Gd ion, at different applied temperatures between 0.4 to 21 K. Open circles represent the total magnetic moment per ion, *m*_TOT_(*H*), determined from XMCD measurements at *T* = 4 K. Bold circle: *m*_TOT_ value obtained from sum rules at 6 T, 4 K. Solid lines represent the theoretical Brillouin prediction for noninteracting Gd­(III) ions with *g* = 2 and no anisotropy (*D* = 0). Red dashed line: *Phi* simulation of the 0.4 K curve using ab initio-calculated parameters. (c) Normalized, background-subtracted XAS and (d) XMCD spectra measured for ***m*** **CB-Gd** at Gd M_4,5_ edges, at 6 T and 4 K.

The field-dependence of the magnetization curves of ***m*** **CB-Gd** at different temperatures are shown in Figure 2b. The *M*(*H*) measured between 1.8 and 21 K are well fit by the theoretical Brillouin prediction for noninteracting Gd­(III) ions with *S* = 7/2, *g* = 2. At very low temperatures, 0.4 K, the magnetization saturates at 7 μ_B_ at 5 T, but the *M*(*H*) curve falls below the Brillouin prediction, indicating the presence of weak AF interactions and/or anisotropy. Notably, the experimental data at 0.4 K could be well fit within *Phi* code,^70^ using the single-ion anisotropy (*D*_i_) and orthorhombic (*E*_i_) parameters for the three distinct Gd­(*i*) sites, along with the interaction constants (*J*_ij_) calculated by ab initio methods (*vide infra*, Table Figure 5a). The same model also satisfactorily fits the χ*T*(*T*) data (Figure 2a, red line).

To further characterize the material, ***m*** **CB-Gd** was analyzed using X-ray absorption spectroscopy (XAS) and X-ray magnetic circular dichroism (XMCD). Figure 2c,d shows the XAS and XMCD spectra measured at 4 K under a magnetic field of 6 T at the Gd M_4,5_ edges. The XAS spectral features are characteristic of Gd­(III), with the M_5_ edge displaying a shoulder at 1182.8 eV, a main peak at 1185.1 and two satellites at 1189.4 and 1192.3 eV. The M_4_ edge exhibits two peaks at 1214.2 and 1215.8 eV, along with a shoulder at 1219.2 eV. We determined the orbital (*m*_L_), spin (*m*_s_) and total magnetic moment (*m*_TOT_ = *m*_L_ + *m*_s_) per Gd­(III) ion in the sample from the XAS-XMCD spectra using the corrected sum rules for lanthanides,^24,25^ yielding *m*_L_ = 0.16 ± 0.01 μ_B_/ion, *m*_S_ = 6.47 ± 0.25 μ_B_/ion and *m*_TOT_ = 6.61 ± 0.4 μ_B_/ion. The field-dependence of the total magnetic moment, *m*_TOT_(*H*), was obtained by following the intensity of the XMCD­(*H*) peak at the M_5_ edge between −6 and 6 T, and scaling the curve with the value of *m*_TOT_ at 6 T. As shown in Figure 2b, the resulting curve agrees well with the Brillouin expectation at 4 K.

The exceptional hydrophobic properties of carborane linkers, along with the stability of our material in both water and organic solvents, enable the preparation of suspensions, facilitating the deposition of the material onto various technical surfaces.^56^ For this study, a methanol suspension of ***m*** **CB-Gd** was prepared and drop-casted onto a Si substrate. Optical images show intact crystallites of ***m*** **CB-Gd** deposited on the Si substrate (Figure S2a). The XAS and XMCD spectra of the on-surface deposited ***m*** **CB-Gd**/Si, shown in Figure S2b,c, exhibit the same features as the bulk material. The spin, orbital, and total magnetic moments at 6 T, as well as the *m*_TOT_(*H*) curve, match the expected values for the sample at *T* = 4.6 K (Figure S2d). These results confirm that the magnetic properties of the ***m*** **CB-Gd** MOF are preserved after methanol-dispersion and surface deposition.

The dynamic magnetic properties of ***m*** **CB-Gd** were characterized by ac susceptibility measurements. Figure 3a shows the out-of-phase component of the susceptibility as a function of the frequency, χ”(*f*, *T*), at a constant applied magnetic field (μ_0_ *H* = 0.2 T) and temperatures between 1.8 and 20 K, while Figure 3b shows χ”(*f*, *H*) curves at a fixed temperature (*T* = 2.0 K) and varying magnetic fields in the 0–1 T range. From these data, three distinct spin relaxation processes are identified, with relaxation times labeled τ_1_, τ_2_ and τ_3_. Figure 3c,d illustrate the dependence of the relaxation time on the inverse temperature, τ­(1/*T*), and the magnetic field, τ­(*H*), for each process.

**Figure 3:**
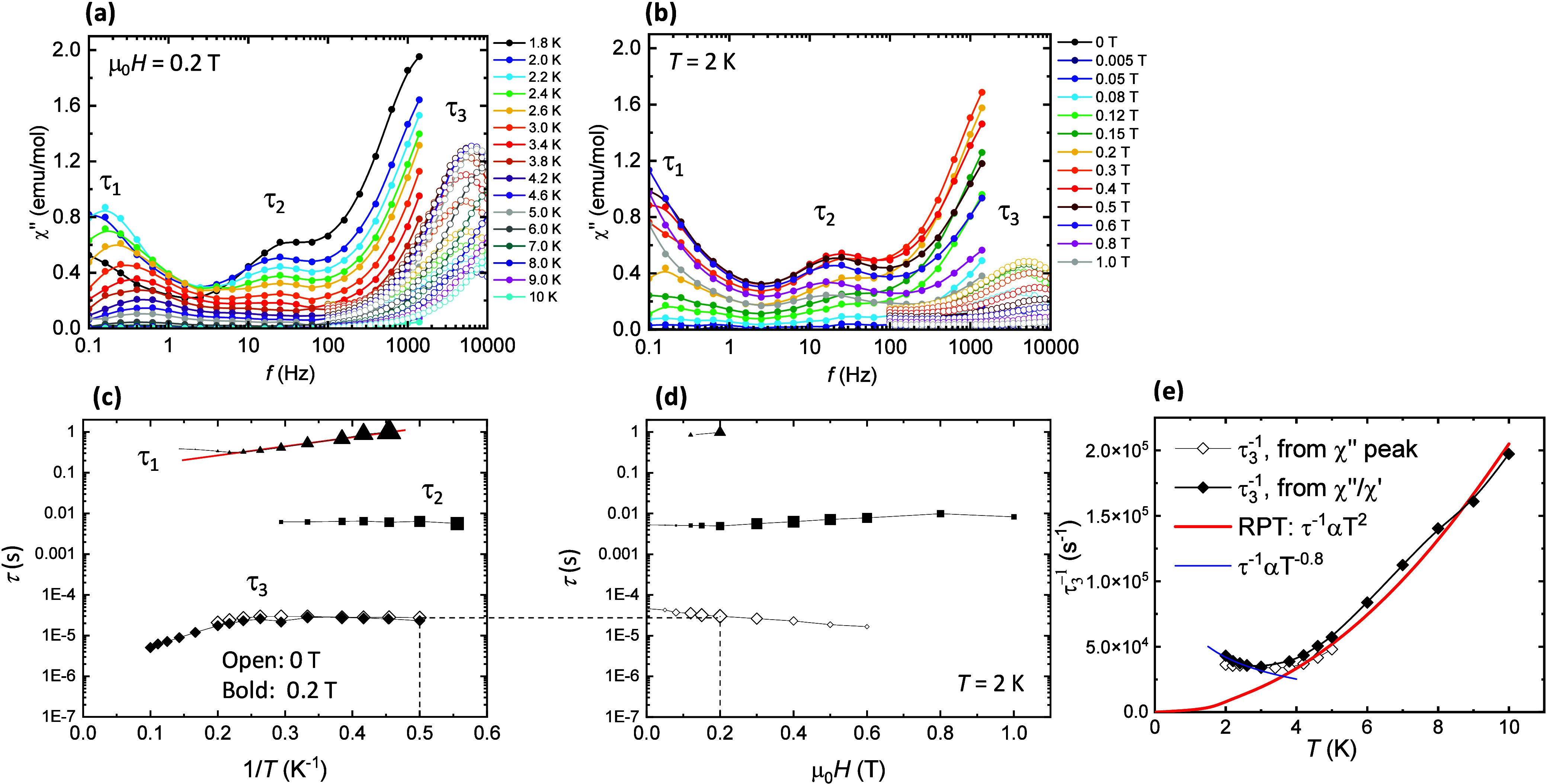
Spin–lattice magnetic relaxation of ***m*** **CB-Gd.** Top: Imaginary component of the ac susceptibility as a function of the frequency, χ”(*f*), of ***m*** **CB-Gd** MOF, (a) at μ_0_ *H* = 0.2 T and different temperatures, and (b) at *T* = 2 K under different applied magnetic fields. Data in the 0.1–10^3^ Hz range (solid symbols) were obtained using a SQUID magnetometer, while data in the 10^2^–10^4^ Hz range (open symbols) were acquired with a PPMS. Bottom: (c) Magnetic relaxation time τ as a function of the inverse temperature at μ_0_ *H* = 0.2 T, and (d) as a function of the applied magnetic field at *T* = 2 K, for the different relaxation processes observed; (e) temperature dependence of the relaxation rate τ_3_^–1^(*T*): fit to a power-law τ_3_^–1^ = *KT* ^2^, where *K* = 2050 s^–1^ K^–2^, between 4–10 K attributed to RPT (red line), and reciprocating thermal behavior, τ_3_^–1^ α *T* ^–0.8^, below 3 K (blue line).

The relaxational behavior of Gd­(III) differs from that of orthodox single-ion magnets (SIMs), since the anisotropy energy due to zero-field-splitting (ZFS) in Gd is small, Δ_ZFS_/*k*_B_ ≈ |*D*|(*S* ^2^ – 1/4), with *S* = 7/2. In this case, based on the ab initio-calculated anisotropy values for the three distinct Gd­(*i*) sites (Figure 5a), the expected ZFS anisotropy values are Δ_ZFS_/*k*_B_ ≈ 0.99 K (Gd1), 1.42 K (Gd2) and 0.65 K (Gd3). These values are comparable to the Zeeman splitting (Δ_H_/*k*_B_ = 1.9 K for *M*_s_ = 7/2 at μ_0_ *H* = 0.2 T).

The first, slowest relaxation process has a relaxation time on the order of τ_1_ ≈ 0.1 to 1 s. The τ_1_(1/*T*) dependence at 0.2 T was fit to an Arrhenius law, with an activation energy of *U*/*k*_B_ = 5.2 K, consistent with the level splitting. Similar field-induced relaxation processes in Gd compounds have been earlier reported under comparable applied dc fields, such as the cyanoacetate complex {[Gd_2_(CNCH_2_COO)_6_(H_2_O)_4_]·2H_2_O}_*n*_ (*U*/*k*_B_ = 5.5 K at 0.2 T),^71^ or [phen_2_Gd_2_(HCOO)_4_(HCOO)_2–2*x*_(NO_3_)_2*x*_] at 0.275 T.^72^

The second relaxation process (τ_2_ ≈ 0.006 s) is nearly independent of temperature and magnetic field. A cross-relaxation mechanism mediated by spin–spin interactions may be hypothesized; however, the origin of this process remains unclear, and we therefore refrain from making a definitive assignment at this stage.

The third relaxation process (τ_3_) is temperature-dependent, but cannot be attributed to an Orbach mechanism, as the effective energy barrier extracted from the ln τ_3_ vs 1/*T* plot at 10 K (≈12.1 K) is an order of magnitude larger than the known anisotropy barrier. However, the relaxation rate τ_3_^–1^ follows a *T* ^2^ dependence in the 4–10 K range (Figure 3e), consistent with a Resonant Phonon Trapping (RPT) mechanism.^71,73,74^ RPT is a specific type of phonon-bottleneck effect in which phonons resonant with the spin transition energy become trapped near spin centers through repeated emission and reabsorption. These trapped phonons form quasi-coherent states whose energy cannot efficiently dissipate into the thermal reservoir, thereby delaying spin–lattice relaxation. For RPT to occur, two main conditions must be met, both of which are satisfied by ***m*CB-Gd**: (i) The magnetic coupling between spin centers must be weak to prevent energy transfer via collective spin modes. In our case, the estimated Gd–Gd coupling yields *J*_*ij*_/*k*_B_ *T* ≈ 10^–3^–10^–4^ indicating that collective excitations are indeed unlikely; (ii) the wavelength of the trapped phonon must be significantly longer than the average spin separation, i.e., *k*_0_ *d*_av_ ≪ 1. For ***m*** **CB-Gd**, considering Δ_H_/*k*_B_ ≈ 1.9 K at 0.2 T and the Debye approximation, *h*ν_0_ = *c*_s_ *k*_0_ with the typical solid sound velocity of *c*_s_ ≈ 5 × 10^3^ m/s yields, *k*_0_ ≈ 8 × 10^6^ m^–1^ and since the average distance between Gd centers is *d*_av_ ≈ 9.5 Å, considering both intra- and interchain distances, we obtain *k*_0_ *d*_av_ ≈ 7.6 × 10^–3^, fulfilling the condition for RPT. The extracted τ_3_ values also agree with those previously reported for RPT processes in Gd compounds.^71,75^ Although a Raman mechanism can exhibit a τ^–1^ ∝ *T* ^2^ dependence under certain conditions,^76^ it is unlikely here, as the 4–10 K range where τ_3_ is observed lies well below the Debye temperature of ***m*** **CB-Gd**, Θ_D_ ≈ 210 ± 10 K, estimated from specific heat data (see Figure S3).

It is noted that, below 4 K, τ_3_^–1^ increases as temperature decreases, following a τ^–1^ ∝ *T* ^–*k*^ trend (*k* ≈ 0.8). This reciprocating-thermal behavior with *k* ≈ 0.5–1 has been previously observed in other Gd,^55,77^ RE and transition-metal compounds,^78^ and has been tentatively attributed to a secondary solution of the phonon-bottleneck equations.^79^

### Heat Capacity and Magnetocaloric Effect

The thermal properties of ***m*** **CB-Gd** were investigated by heat capacity (HC) measurements across a temperature range of 0.3–30 K under magnetic fields varying from 0 to 3 T (Figure 4a). The magnetic contribution to the specific heat, *C*_m_(*T*, *H*), was determined by subtracting the lattice contribution, *C*_L_ = *AT* ^*n*^ (Figure 4b). At zero field, *C*_m_(*T*) exhibits a broad Schottky-like contribution, induced by the crystal field splitting of the Gd­(III) levels, which shifts to higher temperatures for increasing magnetic fields. No signs of AF ordering were observed down to the lowest measured temperature of 0.35 K. The experimental data are well reproduced by theoretical *C*_m_(*T, H*) curves, calculated with *Phi* code considering the ab initio-derived single-ion *D*, *E* parameters for the three Gd­(*i*) sites and *J*_*ij*_ constants (Table Figure 5a).

**Figure 4:**
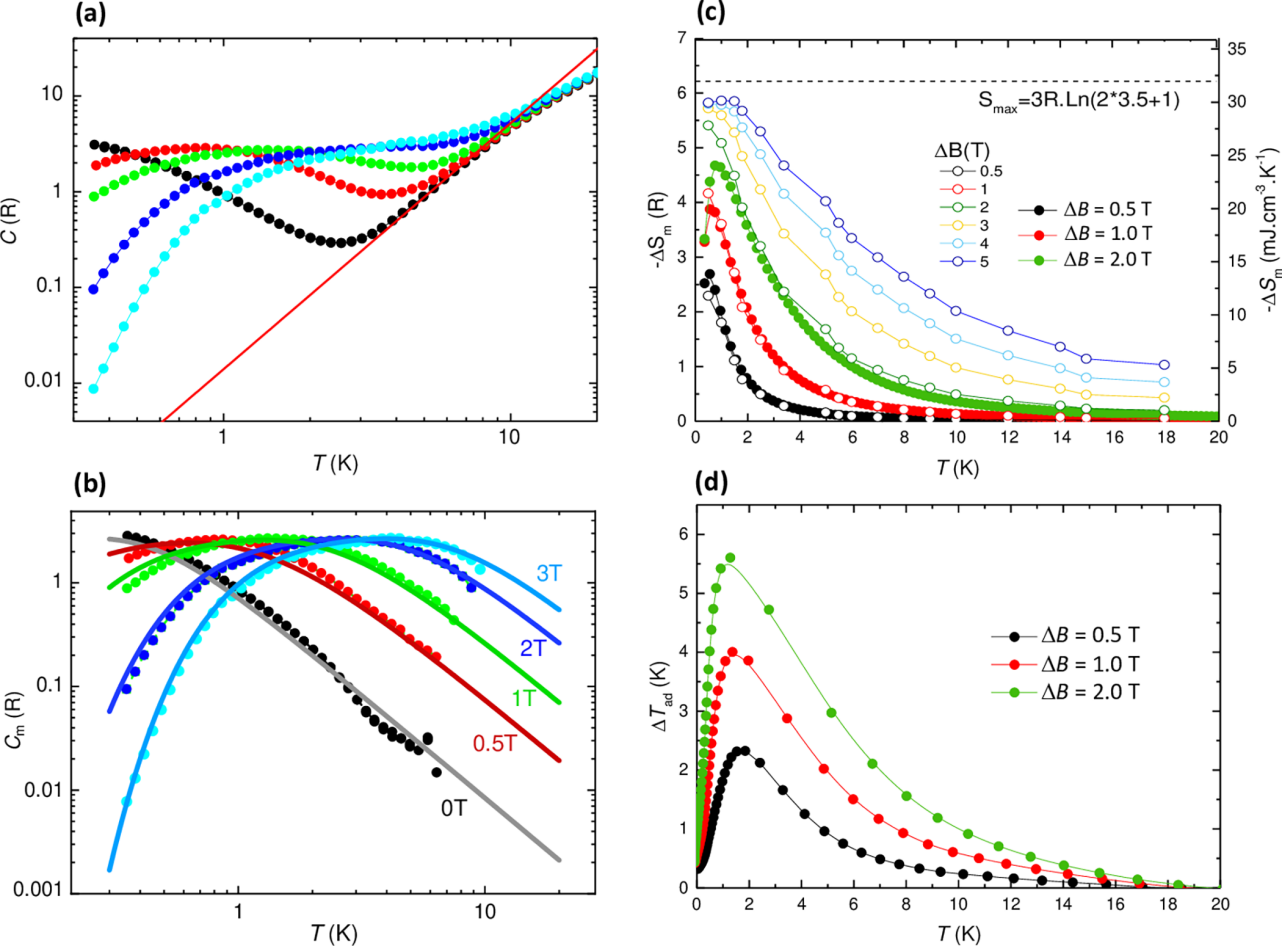
Specific heat and MCE of ***m*** **CB-Gd** quMOF. (a) Specific heat as a function of temperature at the indicated magnetic fields, *C*(*T*,*H*). Red line: lattice contribution, *C*_L_ = A*T* ^n^, with A = 1.4 × 10^–2^ R·K^–2.57^, n = 2.57. (b) Magnetic contribution to the specific heat, *C*_m_(*T*,*H*), and calculated curves using *Phi* code, and ab initio-derived single-ion *D*, *E*, parameters and *J*_*ij*_ coupling constants (Table in Figure 5a). (c) Temperature dependence of the magnetic entropy change, −Δ*S*_m_, obtained from HC measurements for applied magnetic field changes Δ*B* = 0.5, 1, 2 T (bold symbols), and from the *M*(*H*) isotherms shown in Figure 2a, for Δ*B* = 0–5 T (open symbols). The dashed line represents the maximum available entropy for this system, *S*_m_ = 3Rln­(2*S* + 1) = 6.24 R, with *S* = 7/2; (d) temperature dependence of the adiabatic temperature change, Δ*T*_ad_(*T*), obtained from HC for the indicated magnetic field changes Δ*B.*

**Figure 5:**
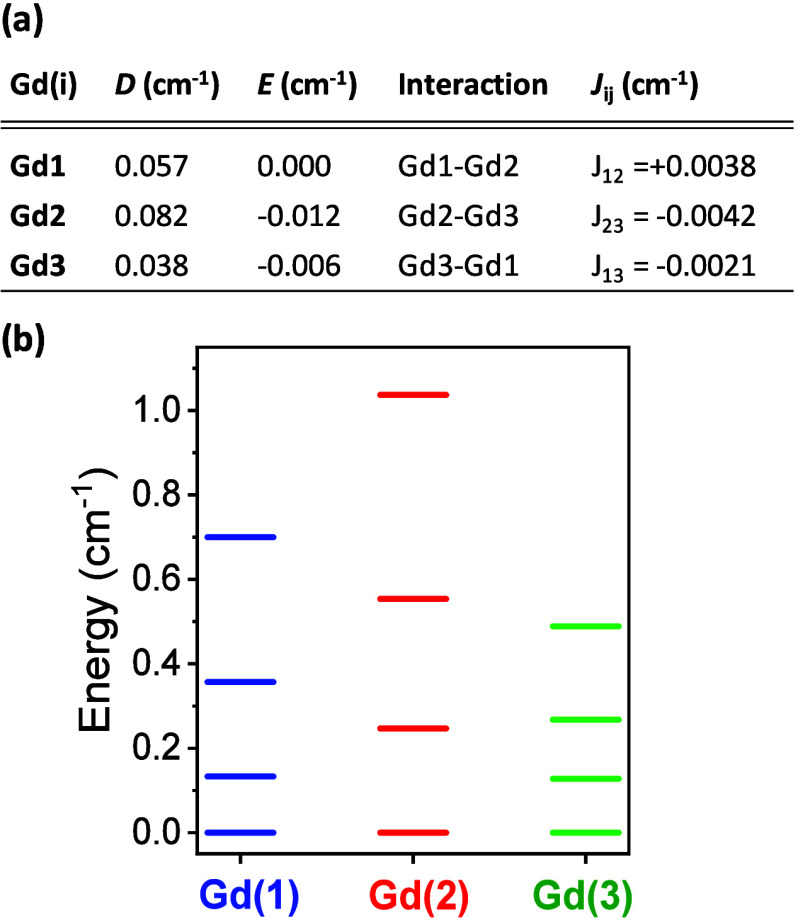
(a) Ab initio-calculated axial (*D*) and orthorhombic (*E*) anisotropy parameters of the ZFS tensor for the three nonequivalent sites for Gd­(III) ions in ***m*** **CB-Gd**, and intrachain n.n. magnetic interactions, within a Hamiltonian ${\hat{H}}_{\mathrm{ex}}=-2\sum J_{\mathrm{ij}}{\vec{S}}_{i}\cdot {\vec{S}}_{j}$ (here *J*_*ij*_ < 0 denotes AF coupling). (b) Calculated energy levels of the ground state for the three distinct Gd­(*i*) coordination sites in zero applied magnetic field.

The magnetocaloric effect (MCE) performance was evaluated from the HC data. Figure 4c shows the magnetic entropy change −Δ*S*_m_(*T*, Δ*B*) for selected field changes Δ*B* = (0 – *B*_f_) = 0.5, 1, 2 T. For practical purposes, it is relevant to discuss results obtained for *B*_f_ = 2 T, as this field can be easily achieved using permanent magnets in magnetic refrigerator applications. At Δ*B* = 2 T, the maximum magnetic entropy change is −Δ*S*_m_^max^ = 4.67 R (24.14 mJ cm^–3^ K^–1^) at *T*_max_ = 0.79 K, which is a 75% of the systems’ total available magnetic entropy, *S*_max_ = 3Rlog­(2*S* + 1) = 6.24 R. Additionally, the adiabatic temperature change Δ*T*_ad_(*T*) = *T*_*f*_ – *T*_i_ was determined (Figure 4d). For Δ*B* = 2 T, the temperature decrease reaches a maximum of Δ*T*_ad_^max^ = 5.61 K at *T* = 1.22 K. The full width at half-maximum of the −Δ*S*_m_ curve is δ*T*_fwhm_ = 2.14 K, yielding a relative cooling power of RCP = −Δ*S*_m_^max^ × δ*T*_fwhm_ = 51.7 mJ cm^–3^. The MCE performance at higher applied magnetic fields, up to 5 T, was further evaluated using magnetization vs field data collected at various temperatures (Figure 2b), applying the Maxwell method.^26^ For Δ*B* = 5 T, the magnetic entropy change reaches 5.88 R, that is, 94.3% of the system’s maximum available entropy (Figure 4c).

### Ab Initio Calculations

The ground state of the free Gd­(III) ion is in a first approximation a pure ^8^S_7/2_ multiplet (*L* = 0, *S* = 7/2), which is spherically symmetric, i.e. isotropic under an external field. However, spin–orbit coupling also produces a small mixing of the ground state octet with the excited sextets or even higher energy multiplets. This mixing, in combination with crystal field effects, gives rise to a zero-field splitting (ZFS) of the ground state multiplet into four Kramers doublets.^80^ The distortion of the coordination atoms around Gd thus determines the actual electronic level scheme. Ab initio calculations, using the CASSCF/RASSI-SO method,^81,82^ were performed to determine the axial and orthorhombic anisotropy parameters, along with the energy levels for the fundamental multiplet, for the three distinct Gd­(*i*) sites in ***m*** **CB-Gd** MOF. The Hamiltonian describing the single-ion level splitting for each Gd­(*i*) under an applied magnetic field is given by

$${\hat{H}}_{i}=D_{i}S_{zi}^{2}+E_{i}(S_{xi}^{2}-S_{yi}^{2})-g\mu_{B}{\vec{S}}_{i}\cdot \vec{B}$$ 1

where *D*_*i*_ and *E*_*i*_ represent for each ion the axial and orthorhombic components of the zero-field splitting tensor producing magnetic anisotropy, and the last term accounts for Zeeman energy term with the *g* ≈ 2. The total Hamiltonian operates on the complete 8-fold basis function of the unperturbed wave function |7/2, *S*_*z*_⟩. The calculated values of *D*_*i*_, *E*_*i*_ are summarized in Figure 5a. For the three coordination environments, Gd is predicted to exhibit planar anisotropy (*D* > 0), indicating that the *z*-axis is the hard magnetization axis. Gd(2) displays the largest anisotropy value (*D*_2_ = 0.082 cm^–1^), differing by 54% from the smallest value calculated for Gd(3). Additionally, we calculated the nearest-neighbor magnetic interactions between the three Gd­(*i*) ions within a chain by applying the Broken Symmetry DFT method, as detailed in the Experimental Section.^83^ The coupling interaction constants, described by an interaction Hamiltonian of the form ${\hat{H}}_{\mathrm{ex}}=-2\sum J_{\mathrm{ij}}{\vec{S}}_{i}\cdot {\vec{S}}_{j}$ are also given in Figure 5a.

At sufficiently low temperatures, the system can be described by the fundamental multiplet formed by the four lowest Kramers doublets (KDs). The ab initio calculated energy levels and components of the gyromagnetic tensor under pseudospin (*g*_x_*, *g*_y_*, *g*_z_*) of these four KDs for the three different Gd­(*i*) coordination sites are summarized in Figure 5 and Table S1.

As demonstrated in previous sections, the experimental magnetization, susceptibility (Figure 2a,b) and heat capacity (Figure 4b) data could be well described by *Phi* calculations within a Hamiltonian encompassing the ab initio-calculated parameters, confirming the validity of the model.

To evaluate the potential of this triple-site Gd_3_ quMOF for Quantum Computing, we calculated its energy level scheme under an applied field using *Phi* software, incorporating the ab initio-derived *D*_*i*_, *E*_*i*_ and *J*_*ij*_ parameters. As shown in Figure 6a, the calculated energy level scheme at 3.6 K, mapped as a function of the magnetic field up to μ_0_ *H =* 1 T, reveals 512 separated states, sufficient to encode up to *N* = 9 qubits. This finding highlights the system’s capacity to address scaling challenges in Quantum Computing.

**Figure 6:**
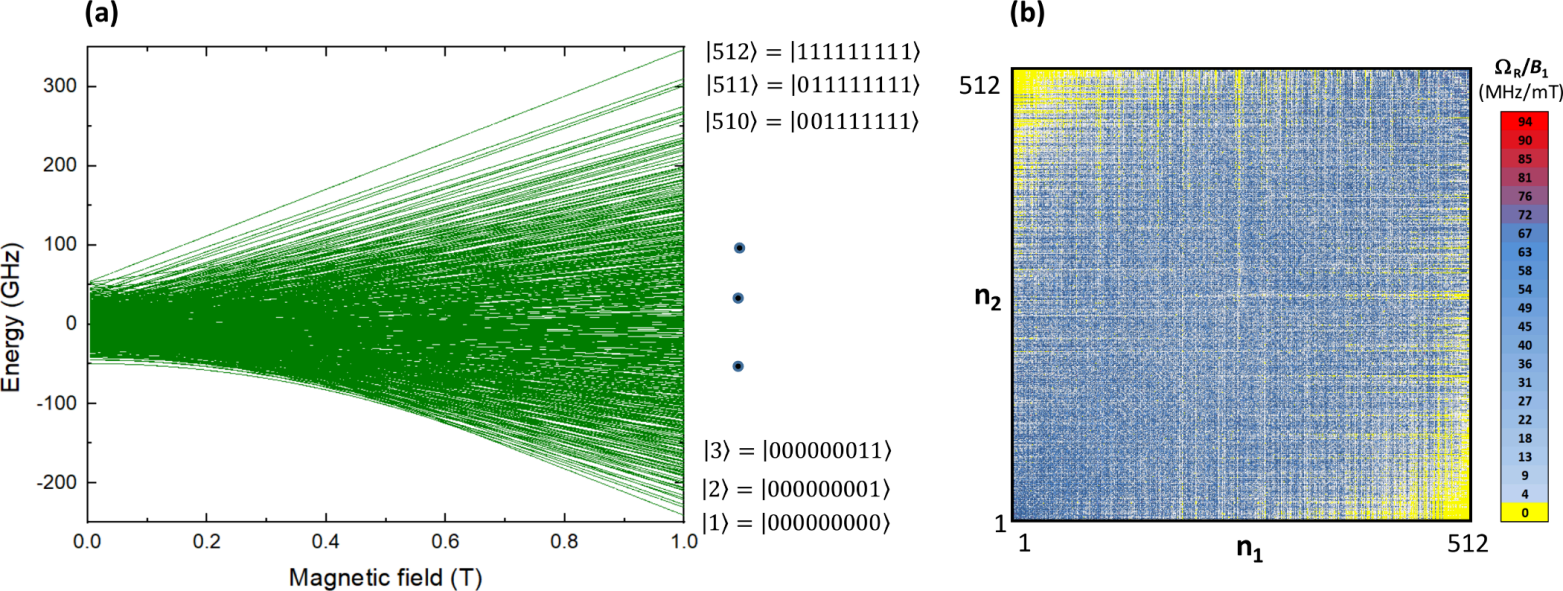
512-level qudit implemented in ***m*** **CB-Gd** quMOF. (a) Energy level scheme calculated using *Phi* suite and ab initio-derived parameters at 3.6 K with the magnetic field applied in the *z*-direction. (b) Color map of the Rabi frequencies induced by *B*_1_-driven resonant transitions between adjacent levels, calculated at 0.6 T (along the *z*-axis).

An important aspect to note is that this quMOF would admit quantum universal operations,^41^ implying that any quantum superposition of the 512-dimension Hilbert space can be generated from any initial state through the application of a sequence of electromagnetic pulses. This capability arises from the existence of allowed transitions between the different spin levels, which can be selectively addressed by tuning the frequency of the pulse or adjusting the external magnetic field. The condition of universality for ***m*** **CB-Gd** quMOF is demonstrated in Figure 6b, where we plot the Rabi frequencies (normalized by the driving field *B*_1_) calculated at an intermediate field 0.6 T linking the different levels, numbered from 1 to 512. The dense map of allowed transitions makes it possible to access any final state by concatenating successive (*n_i_* – *n*_*i*+1_) resonant transitions at different frequencies.

It is noted that the crowding of the states would impede coherent manipulation of this qudit using conventional X-band technology, typically operating at microwave frequencies (∼10 GHz). However, ongoing developments in L-band (1 GHz) EPR^84,85^ may provide the fine spectral resolution needed to resolve these closely spaced transitions. Moreover, emerging broadband spectroscopy instrumentation^86^ offers promising potential to enable tailored multifrequency manipulation of large-dimensional qudits.^87^

Additionally, the magnetically diluted *m*CB-GdY MOFs contain isolated Gd ions in 3 distinct coordination sites, each capable of functioning as an 8-state qudit. To illustrate this, we simulated the field-split energy level scheme for each Gd­(*i*) site using ab initio-derived *E*_*i*_, *D*_*i*_ parameters. Figure 7a–c demonstrates that all three Gd­(*i*) sites exhibit 8 well-separated energy levels, with 7 transitions that would be accessible via standard X-band EPR at 10 GHz. Furthermore, the condition of universality is satisfied for each of the three 8-level qudits, as evidenced by the calculated Rabi frequencies at 0.6 T along the *z*-axis for each Gd­(*i*) ion (Figure 7d–f). Additional data, including calculated Rabi frequencies for other magnetic field orientations and values, are provided in the Supporting Information (S5).

**Figure 7:**
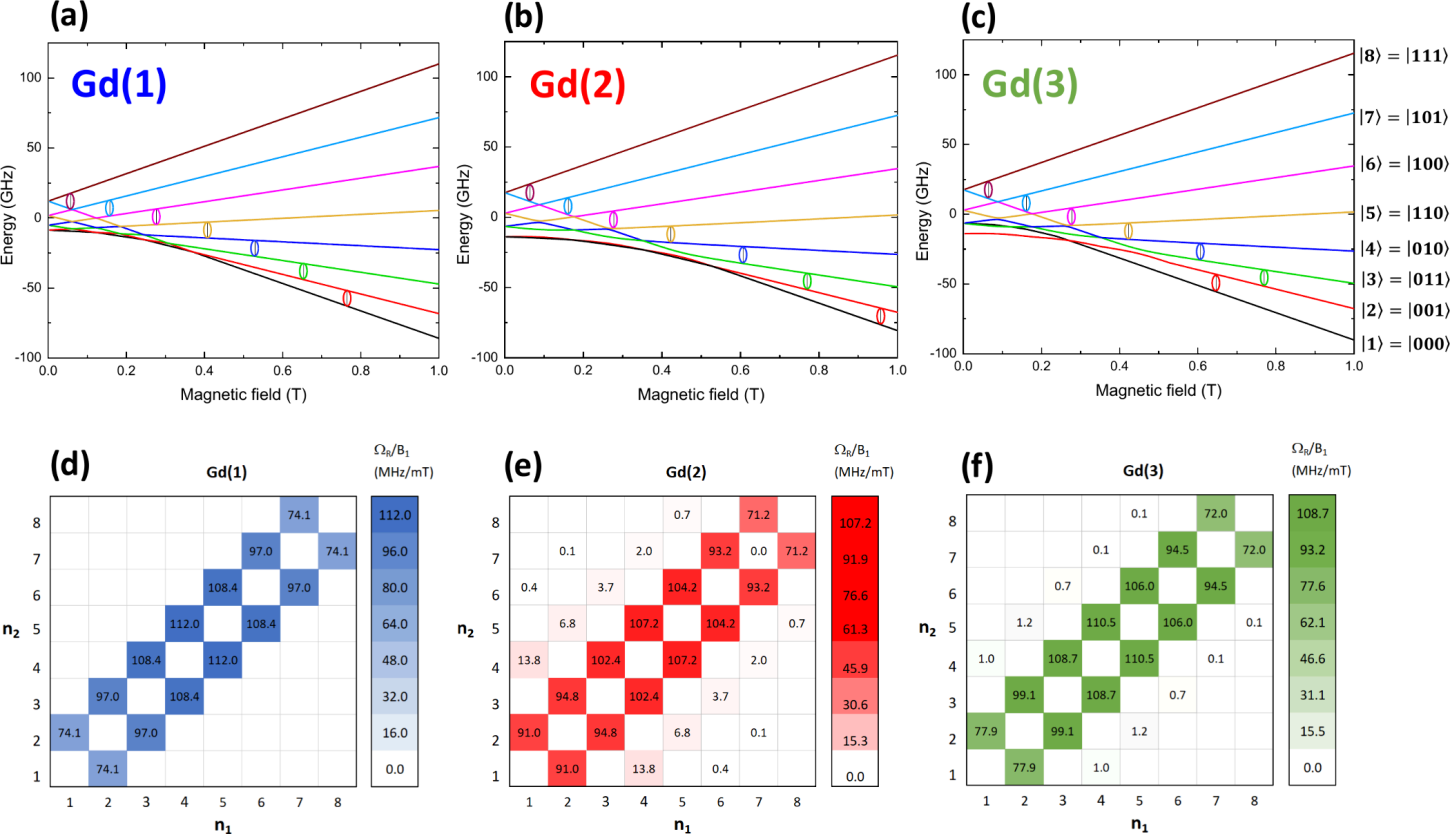
Three isolated 8-level qudits. (Top) Energy level scheme calculated using *Phi* suite and ab initio-derived parameters at 3.6 K with *H*//z for (a) Gd(1), (b) Gd(2), (c) Gd(3) in magnetically diluted *m*CB-Gd_*y*_Y_100–*y*_ MOFs, behaving as 8-level qudits. (Bottom) Rabi frequencies (normalized by *B*_1_) calculated at 0.6 T (along the *z*-axis) for each Gd­(*i*) (d–f).

### Pulsed EPR quMOF Characterization

Pulsed EPR measurements were conducted on two quMOFs with different percentage of magnetic dilution, ***m*** **CB-Gd**_**0.08%**_ and ***m*** **CB-Gd**_**1.76%**_. The echo-detected field-swept (ES-EPR) X-band EPR spectra (9.5 GHz) of the two powdered samples at 6 and 10 K are presented in Figure S8. The X-band continuous wave (CW) EPR spectrum for the most diluted sample (***m*** **CB-Gd**_**0.08%**_) at 3.6 K, derived from the first derivative of the ES-EPR spectrum, is presented in Figure 8e. In this sample, the magnetic interactions between Gd ions are negligible due to the high dilution. The experimental CW-EPR spectrum closely matches the theoretical prediction, which was obtained as a weighted linear combination of the spectra corresponding to the three distinct Gd­(*i*) sites (*i* = 1, 2, 3), shown in Figure 8d. These individual spectra were calculated using *Phi* suite, with zero-field splitting parameters (*D*_*i*_, *E*_*i*_) derived from ab initio (Figure 8a–c). The excellent agreement between the experimental and theoretical spectra demonstrates the reliability of the ab initio-derived parameters.

**Figure 8:**
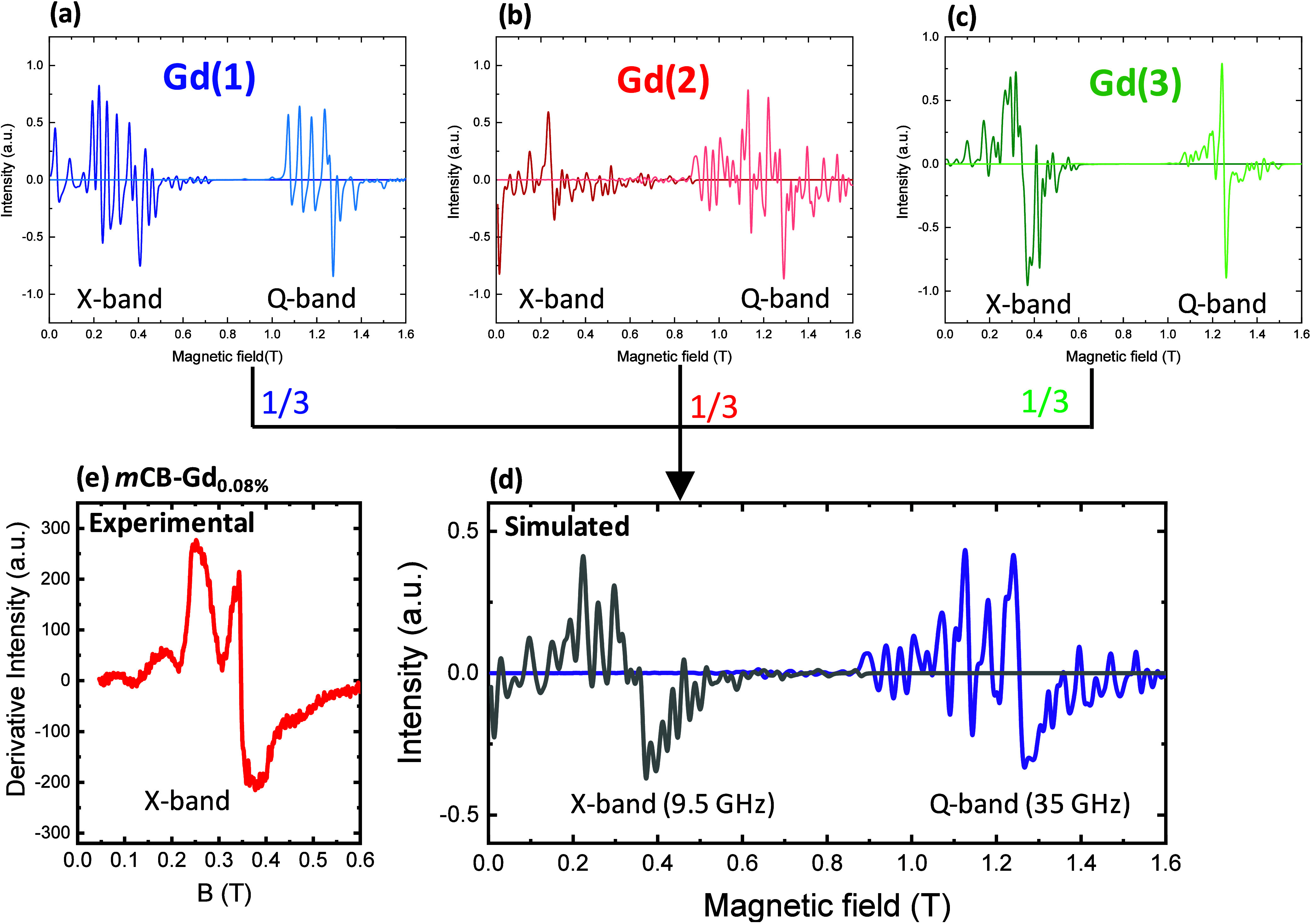
CW-EPR spectra of Gd­(*i*) sites and diluted GdY quMOF. (Top) Continuous-wave EPR (CW-EPR) spectra at X-band (9.5 GHz) and Q-band (35 GHz) at 3.6 K for the three distinct Gd­(*i*) sites in ***m*CB-Gd** quMOF, (a) Gd(1), (b) Gd(2), and (c) Gd(3), calculated using *Phi* with ab initio-derived *D*_*i*_, *E*_*i*_ parameters. (d) Simulated CW-EPR spectra for a magnetically diluted *m*CB-GdY MOF, assuming an equimolar contribution from all three isolated Gd­(*i*) sites. (e) Experimental X-band spectrum for the ***m*** **CB-Gd**_**0.08%**_ sample.

Pulsed EPR experiments were conducted between 3.6 and 50 K on the two magnetically diluted ***m*** **CB-Gd** **_*y*_** (*y* = 0.08%, 1.76%) MOFs to investigate qubit spin dynamics. The spin–lattice relaxation time *T*_1_ was determined using a standard inversion recovery sequence (see Experimental Section). Inversion recovery data were collected at different temperatures within this range, both at the field position corresponding to the maximum of the field-swept echo-detected spectra, as well as at other lateral field positions (Figure S9). Experimental data were fit with a biexponential function, Eq. (2), yielding a short (*T*_1,s_) and a long (*T*_1,L_) spin–lattice relaxation times.

The phase-memory time *T*_m_ was determined using a Hahn spin echo decay sequence, and the echo intensity data was fit to a single-exponential function, Eq. (3). *T*_1_ and *T*_m_ were measured at the field position corresponding to the maximum (*B* = 346 mT) of the field-swept echo-detected spectra across 3.6–40 K for ***m*** **CB-Gd**_**0.08%**_ and 6–10 K for ***m*** **CB-Gd**_**1.76%**_. Experimental data and corresponding fit parameters for *T*_1,s_, *T*_1,L_ are provided in Figure S9/Table S3 (***m*** **CB-Gd**_**0.08%**_) and Figure S10/Table S4 (***m*** **CB-Gd**_**1.76%**_), while those for *T*_m_ are in Figure S11/Table S5 (***m*** **CB-Gd**_**0.08%**_) and Figure S12/Table S6 (***m*** **CB-Gd**_**1.76%**_).

The temperature dependence of *T*_1_ and *T*_m_ for both quMOFs is shown in Figure 9a. As expected, the most diluted sample (***m*** **CB-Gd**_**0.08%**_) displays longer relaxation times. At the lowest tested temperature (3.6 K), the spin–lattice relaxation times reach *T*_1,s_ = 33 μs and *T*_1,L_ = 256 μs. The phase memory time is *T*_m_ = 0.7 μs, comparable to previously reported values for other Gd-based quMOFs.^53^ *T*_1_ displays a stronger temperature dependence, following a stronger power-law dependence (*T*_1L_ α *T* ^2.08^, *T*_1S_ α *T* ^1.68^), compared to *T*_m_ α *T* ^0.77^. Although *T*_m_ remains significantly shorter than *T*_1_ across the full temperature range, an analysis of the decoherence contributions —considering spin–lattice relaxation, electronic spectral diffusion (SD), electron spin-flop, nuclear SD and instantaneous diffusion (ID) — reveals that *T*_m_ is still indirectly limited by *T*_1_ through the dominant electronics SD contribution (see details in Figure S13).

**Figure 9:**
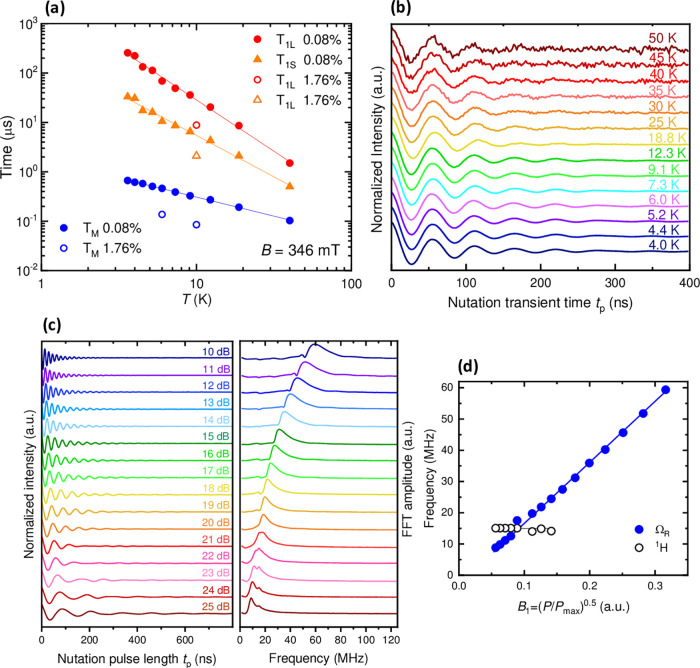
Pulsed EPR of *m*CB-GdY quMOFs. (a) Temperature-dependence of *T*_1_ and *T*_m_ obtained at 346 mT for magnetically diluted samples ***m*** **CB-Gd**_**0.08%**_ and ***m*** **CB-Gd**_**1.76%.**_ (b) Rabi oscillations for ***m*** **CB-Gd**_**0.08%**_ at 346 mT for different temperatures between 4–50 K. (c) Nutation experiments on ***m*** **CB-Gd**_**0.08%**_ at 3.6 K for different *M*_w_ attenuation values in dB, and (right) Fast Fourier Transform (FFT). (d) Rabi frequencies obtained from FFT as a function of the microwave field *B*_1_.

Nutation experiments were performed on the most diluted quMOF, ***m*** **CB-Gd**_**0.08%**_. These measurements were conducted at the maximum of the ES-EPR spectrum (346 mT) over a range of MW powers (10–25 dB) and temperatures (3.6–50 K). As shown in Figure 9b, nutation experiments reveal clear Rabi oscillations at all temperatures up to 50 K.

For measurements at 3.6 K, a decrease in the nutation frequency with increasing attenuation (i.e., lower MW field *B*_1_) was observed (Figure 9c). The Fast Fourier Transform (FFT) of these data reveals a Rabi oscillation frequency (Ω_R_), which increases linearly with *B*_1_ (Figure 9d), demonstrating feasible coherent manipulation via X-band EPR. For the maximum *B*_1_ (lowest attenuation value, 10 dB), the Rabi frequency is Ω_R_ ≈ 60 MHz. Additionally, a field-independent peak is observed at 15.07 MHz, assigned to the Larmor frequency of ^1^H.

To better interpret these results, we calculated the expected normalized Rabi frequencies (Ω_R_/*B*_1_, in MHz/mT) between adjacent levels (*n_i_* – *n_j_*) for the three Gd­(*i*) ions under the experimental field of *B* = 346 mT, applied along both the *z* and *y* directions (Figure S6). The Ω_R_/*B*_1_ maps reveal that for the principal −1/2 ↔ +1/2 transition (*n*_1_ = 4 → *n*_2_ = 5) at the Gd(1) and Gd(3) sites with *H*//*z*, the expected values are Ω_R_/*B*_1_ ≈ 112–105.7 MHz/mT. Assuming *B*_1_ ≈ 0.54 mT at 10 dB in our setup (consistent with the 0.2–0.6 mT range reported for the same equipment under similar conditions using free-electron systems), the theoretical Rabi frequency is Ω_R_ ≈ 60.4–57.1 MHz (Figure S6), in good agreement with the experimental value.

## Conclusions

The reported Gd­(III) carborane-based metal–organic framework {[(Gd)_3_(*m*CB-L)_4_(NO_3_)­(DMF)_*x*_]_*n*_·Solv}, featuring three distinct coordination sites, represents a versatile multifunctional molecular material with exciting potential for Quantum Computing and magnetic refrigeration applications.

The static magneto-thermal properties of Gd­(III) MOF, measured down to 0.35 K, were accurately modeled considering the site-specific anisotropy parameters and coupling interactions determined by ab initio calculations. Ac susceptibility measurements reveal spin–lattice magnetic relaxation associated to three mechanisms among which Rapid Phonon Trapping. Moreover, Gd­(III) MOF exhibits a notable magnetocaloric effect (MCE). In fact, leveraging the optimal MCE of Gd­(III) and the ability of carborane linkers to form multimetallic frameworks, we recently demonstrated the creation of multifunctional, “self-refrigerated” GdLn (Ln = Dy, Tb, Eu, Eu/Tb) mixed^69^ MOFs, further broadening the application scope. Interestingly, the magnetic properties remain unaltered upon surface deposition, as demonstrated by XAS-XMCD, highlighting its potential for device integration and on-chip cooling technologies.

The use of MOFs with distinct Ln­(III) coordination environments provides a new strategy to tackle the scalability challenge in Quantum Computing. The reported ***m*** **CB-Gd** carborane-based quMOF, with three distinct coordination sites for Gd, represents a large dimensionality 512-state qudit, which, under appropriate conditions can simulate either a 9-qubit processor, or three decoupled 8-level qudits, offering significant potential for quantum operations. Qubit characterization via pulsed EPR in a magnetically diluted (y = 0.08%) GdY MOF revealed a phase-memory time of *T*_m_ = 0.7 μs at 3.6 K and robust Rabi oscillations up to 50 K. These findings position this Gd quMOF as a promising multifunctional platform capable of supporting quantum operations across a broad temperature range.

Looking ahead, future research should focus on achieving coherent manipulation of this 512-qudit quMOF through advanced EPR instrumentation. Additionally, the presence of multiple, distinct 8-level qudits within the framework provides an opportunity to explore parallel quantum computing, potentially enabling more complex quantum algorithms and enhanced computational power.

## Experimental Section

### Synthesis

All chemicals were of reagent-grade quality. They were purchased from commercial sources and used as received. 1,7-di­(4-carboxyphenyl)-1,7-dicarba-*closo*-dodecaborane ligand (*m*CBH_2_L) was synthesized by a slight modification of a literature procedure.^88^ {[(Gd)_3_(*m*CB-L)_4_(NO_3_)­(DMF)_*x*_]_*n*_·Solv} (***m*** **CB-Gd**) was synthesized following a previously described procedure.^67^ The two magnetically diluted MOFs, {[(Gd_*y*_Y_100–*y*_)_3_(*m*CB-L)_4_(NO_3_)­(DMF)_*x*_]_*n*_·Solv}, with y = 0.08 (***m*** **CB-Gd**_**0.08%**_) and *y* = 1.76 (***m*** **CB-Gd**_**1.76%**_), were synthesized following the same procedure but using stock methanol solutions of Y­(NO_3_)_3_·6H_2_O (0.04 M) and Gd­(NO_3_)_3_·6H_2_O (0.01 M) instead of the solid salts for greater accuracy. ***m*** **CB-Gd**_**0.08%**_, ICP­(wt %): Gd(0.02 ± 0.005), Y(13.7 ± 0.05). ***m*** **CB-Gd**_**1.76%,**_ ICP­(wt %): Gd(0.37 ± 0.005), Y(11.7 ± 0.05). The samples were only air-dried and no activation was carried out prior to magnetometry.

### Structural Characterization

Powder X-ray Diffraction (PXRD) was recorded at room temperature on a Siemens D-5000 diffractometer with Cu Kα radiation (λ = 1.5418 Å, 35 kV, 35 mA, increment = 0.02°). Inductively Coupled Plasma–Mass Spectrometry (ICP-MS) measurements for GdY mixed MOFs were carried out in an Agilent ICP-MS 7700x apparatus.

### Magneto-Thermal Characterization

Dc magnetometry measurements in the temperature range 1.8 to 300 K were collected using a Quantum Design MPMS SQUID equipped with a 5 T magnet. Experiments were conducted on a powder sample of ***m*** **CB-Gd** embedded in Daphne oil to prevent grain orientation. Ac susceptibility measurements in the range between 1.8–9.0 K, at μ_0_ *H*_ac_ = 4.1 × 10^–4^ T, μ_0_ *H*_dc_ = 0–2.5 T in the range of frequencies between *f* = 0.1–1000 Hz were determined in the same SQUID magnetometer. Furthermore, measurements in the frequency range between *f* = 100–10000 Hz were carried out in a Quantum Design PPMS susceptometer. Additional *M*(*H*) measurements at 0.4 K were conducted in a Quantum Design MPMS3 magnetometer equipped with a ^3^He refrigerator.

Heat capacity measurements as a function of the temperature were performed between 0.3–300 K at different applied fields between 0–3 T on a pressed powder pellet fixed with Apiezon N grease, using a Quantum Design PPMS equipped with a ^3^He refrigerator.

### XAS-XMCD

X-ray absorption spectroscopy (XAS) and X-ray magnetic circular dichroism (XMCD) experiments across the M_4,5_ edge of Gd were performed at BOREAS beamline in ALBA synchrotron. For ***m*** **CB-Gd** a powdered sample was crashed on an indium foil and placed at the top of the coldfinger. Measurements were performed at 4.0 K ± 0.5 K. In addition, a methanol suspension of ***m*** **CB-Gd** crystallites was deposited by drop-casting on a Si substrate. Optical microscopy images of the deposited ***m*CB-Gd**/Si crystallites were collected by a Olympus BX51 Microscope. For XAS-XMCD measurements the sample was glued with silver paint to the sample holder, and sample temperature was 4.6 K ± 0.5 K. All spectra were recorded using Total Electron Yield (TEY) detection mode, with a 90% circularly polarized light. The XMCD (μ^–^ – μ^+^) and XAS (μ^+^ + μ^–^/2) spectra at 6 T were determined from eight X-ray absorption spectra measured under right-handed (μ^+^) and left-handed (μ^–^) circular polarizations. XMCD­(*H*) cycles were performed by following the resonant M_5_ peak while sweeping the magnetic field between 6 and −6 T at a rate of 2 T/min.

### Pulsed EPR

Pulsed Electron Paramagnetic Resonance (EPR) experiments were performed with a Bruker ELEXSYS 580 spectrometer operating at X-band (ca. 9.7 GHz) at the Instituto de Nanociencia y Materiales de Aragón (INMA). The spectrometer was equipped with a Bruker MD5 resonator fitted in a helium gas-flow cryostat from Oxford Instruments. Temperature control was exerted using a MercuryTC device, also from Oxford Instruments. Echo-Detected EPR experiments were obtained by acquiring the integrated intensity of a Hahn echo while sweeping the external magnetic field. For representation purposes, some of these absorption-like spectra were pseudomodulated in silico and compared to the corresponding simulation. EPR spectra were calculated using *Phi* suite, using zero-field splitting parameters for each Gd­(*i* = 1, 2, 3) site that were obtained from the ab initio calculations.

Spin–lattice relaxation time *T*_1_ was determined with a standard inversion recovery sequence (π/2 – *t* – π/2 – τ – π – τ – echo) for variable *t* and pulse lengths *t*_π/2_ = 16 ns and *t*_π_ = 32 ns. Echo inversion recovery data were collected at different temperatures between 3.6 and 50 K at the maximum of the field-swept EPR spectrum (*B* = 346 mT for ***m*** **CB-Gd**_**0.08%**_, *B* = 358 mT for ***m*** **CB-Gd**_**1.76%**_), as well as at other lateral field positions. The experimental data were fit with the biexponential function:

$$I(t)=A\exp(-t/T_{1,L})+B\exp(-t/T_{1,S})+y_{0}$$ 2

where *T*_1,s_ and *T*_1,L_ stand for a short and long spin–lattice relaxation times, and *A* and *B* are negative coefficients.

The phase memory time *T*_m_ was determined using a Hahn spin echo decay sequence (π/2 – τ – π – τ – echo), fitting the echo decay time-trace to the following equation:

$$I(2\tau )=A\exp(-2\tau /T_{m})+y_{0}$$ 3

We note that, because our Hahn-echo experiments probe an ensemble of spins, the observed echo decay constant corresponds to the phase-memory time *T*_m_, which encompasses both homogeneous spin–spin relaxation (*T*_2_) and additional dephasing processes (spectral diffusion, instantaneous diffusion, and unresolved inhomogeneities). Nutation experiments were performed for ***m*** **CB-Gd**_**0.08%**_ using the pulse sequence *t*_p_ – *T* – π/2 – *t* – π – *t* – echo, where *t*_p_ is the nutation pulse of variable length, which was gradually increased during the experiment between 0 to 0.9 μs in steps of 2 ns, and *T* = 112 ns. Several nutation experiments were performed at different power of the nutation pulse, while the detection pulses π/2 and π were optimized for each microwave power, and a two-step phase cycle was applied on the π/2 detection pulse. Integration was performed over the whole echo to eliminate off-resonance contributions.

### Ab Initio Calculations

The single-ion magnetic anisotropy parameters and Gd­(III) energy levels were computed using the CASSCF/RASSI-SO multireference wave function method^81,82^ as implemented in the Molcas 8.6 package.^89^ The atomic positions for the molecular fragments in the calculations were derived from the X-ray crystal structure of the isostructural Tb­(III) derivative. The cluster models include the Gd­(III) ion under study, two paramagnetic Y­(III) ions placed at the positions of the two nearest Gd­(III) ions, and all ligands surrounding the Gd­(III) ion. In these models, the carborane linkers were replaced with benzoate ions. Additionally, the ligands coordinating to the two closest Gd­(III) ions were also included, with DMF ions simplified to formaldehyde and carborane linkers to formate ions. All atoms were represented using basis sets of atomic natural orbitals from the ANO RCC library.^90^ The following basis sets were employed: VQZP for the Gd ion, VTZP for the O, C, and N atoms within the first three coordination shells around the Gd ion, and VDZ for all other atoms.

A CASSCF active space with 7 electrons in the 7 Gd 4f orbitals (CAS­(7,7)) was used to compute 1 octet and 48 sextets. The RASSI-SO calculations were performed using the AMFI approximation^91^ for spin–orbit coupling, with relativistic effects treated using the DKH2 Douglas-Kroll Hamiltonian.^92^ Finally, the SINGLE_ANISO module^89^ was employed to calculate the effective spin Hamiltonian parameters for the Gd­(III) ground state multiplet.

The magnetic coupling constants were calculated using the Yamaguchi′s DFT Broken-Symmetry approach,^83^ as implemented in the Orca 6.0 package.^93,94^ The molecular fragments consisted of the two interacting Gd­(III) ions, with the nearest Gd­(III) ions replaced by Y­(III) ions, along with all ligands coordinated to these four Gd­(III) ions, applying the same simplifications as in the CASSCF/RASSI-SO calculations. The DFT calculations were carried out using the B3LYP^95,96^ functional and the following basis sets: SARC-DKH-TZVP^97^ for the Gd­(III) and Y­(III) ions, and DKH-def2-TZVP^98^ for all other atoms, both of them including scalar relativistic effects. To speed up the calculations the SARC/J auxiliary basis^97^ along with the resolution of identity (RI)^99^ and the chain-of-spheres (COSX)^100^ approximations were used.

## Supplementary Material



## Notes

S1, additional structural characterization; S2, XAS-XMCD of surface-deposited *m*CB-Gd MOF; S3, Debye temperature estimation; S4, ab initio calculations; S5, calculation of Rabi frequencies; and S6, EPR spectroscopy (PDF)
