# Supplementary material for: A Triple-Site Gd3 Carborane Metal–Organic Framework toward Scalable Quantum Computing
Source: ACS Appl Mater Interfaces. 2025 Jun 12;17(29):42082–95. doi: 10.1021/acsami.5c06002 (PMC12291048; doi:10.1021/acsami.5c06002)
Supplement: Supplementary file 1 [file am5c06002_si_001.pdf]

Supporting Information

A Triple-Site Gd_3 Carborane Metal-Organic Framework Toward Scalable Quantum Computing

Elena Bartolomé,[a]* Xiao-Bao Li,[a] Ana Arauzo,[b] Javier Luzón,[c] Inés García-Rubio,[b] José Giner Planas[a]*

[a] Institut de Ciència de Materials de Barcelona (ICMAB), Consejo Superior de Investigaciones Científicas (CSIC), Campus UAB, 08193-Bellaterra, Barcelona, Spain

[b] Instituto de Nanociencia y Materiales de Aragón (INMA), CSIC-Universidad de Zaragoza, and Departamento de Física de la Materia Condensada, 50009 Zaragoza, Spain

[c] Centro Universitario de Defensa (CUD), Carretera de Huesca s/n, 50090-Zaragoza, Spain

*e-mail: ebartolome@icmab.es, jginerplanas@icmab.es

S1. Additional structural characterization **p. 2**

S2. XAS-XMCD of surface-deposited *m*CB-Gd MOF **p. 3**

S3. Debye temperature estimation **p.4**

S4. *Ab initio* calculations **p. 5**

S5. Calculation of Rabi frequencies **p. 6**

S6. EPR spectroscopy **p. 10**

S1. Additional structural characterization

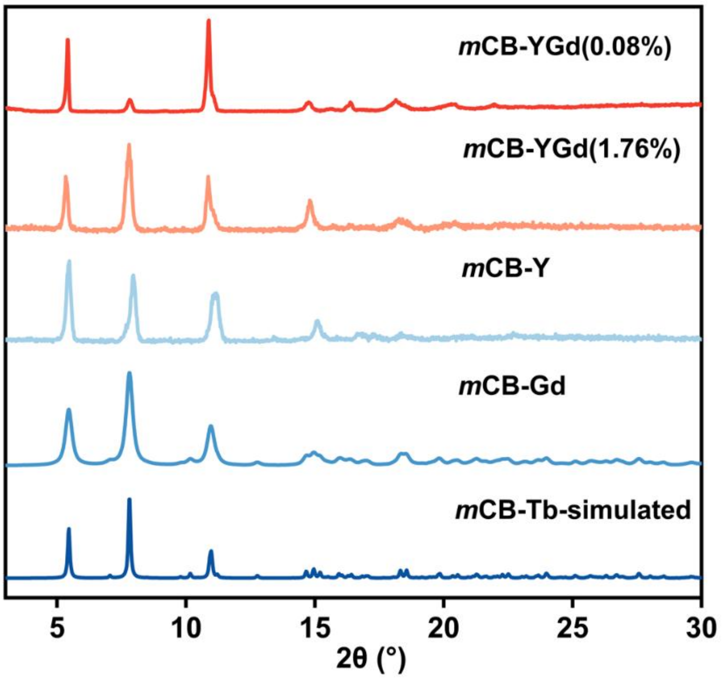

Figure S1. Powder X-ray diffraction (PXRD) spectra for the carborane-based homonuclear ***m*CB-Ln** (Ln = Gd, Y), two magnetically diluted GdY-quMOFs with different percentage of dilution, ***m*CB-Gd$_{0.08\%}$** and ***m*CB-Gd$_{1.76\%}$** and simulated *m*CB-Tb (dark blue), demonstrating phase purity and isostructurality.

S2. XAS-XMCD of surface-deposited *m*CB-Gd MOF

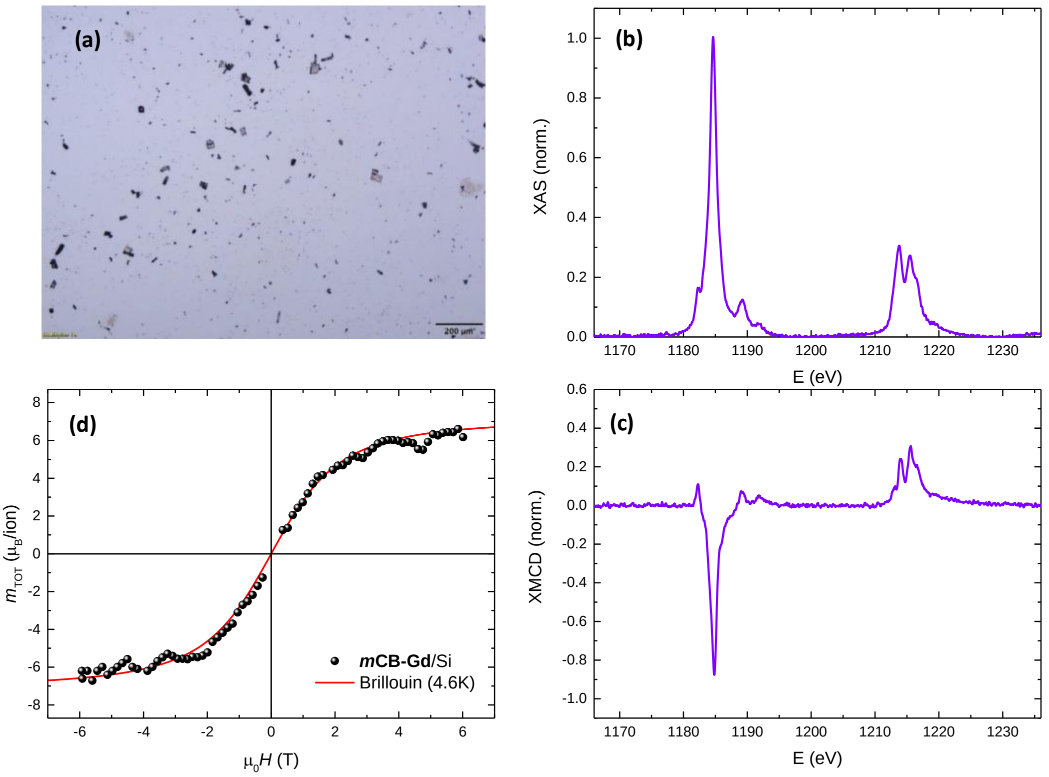

Figure S2. (a) Optical microscopy photograph of ***m*CB-Gd** MOF crystallites deposited on silicon by drop-casting; (b) normalized, background-subtracted XAS and(c) XMCD spectra at the $M_{4,5}$ edge of Gd(III) measured at 6 T and 4.6 K. Application of the sum rules yield values of the orbital, spin and total magnetic moments of m_L = 0.17 μ_B/ion, m_S = 6.20 μ_B/ion and m_{TOT} = 6.37 μ_B/ion, respectively; (d) field-dependence of the total magnetic moment, $m_{tot}(H)$, obtained from XMCD(H), and theoretical expected $m_{TOT}(H)$ curve for a Brillouin function with S = 7/2, g = 2 at 4.6 K.

S3. Debye temperature estimation

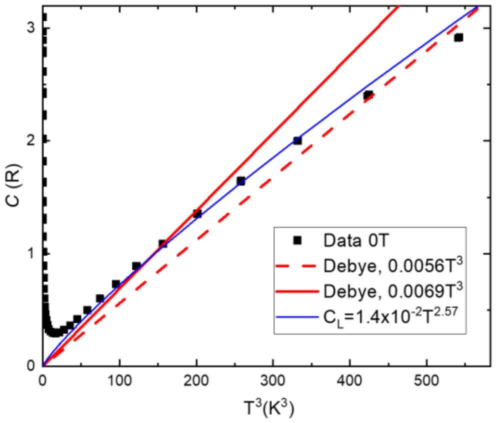

Figure S3. Plot of the molar specific heat (C/R, where R is the gas constant) versus T^3 at 0 T for ***m*CB-Gd**. Within the Debye model, the low-temperature lattice contribution to the specific heat is approximated as $C_L/R = (12\pi^4 r/5)(T/\Theta_D)^3$, where *r* is the number of atoms per formula unit, *T* is the temperature, and Θ_D is the Debye temperature. The data for ***m*CB-Gd** slightly deviates from ideal behavior (blue line: $C_L = AT^{2.57}$, $A=1.4 \times 10^{-2} R.K^{-2.57}$). Nonetheless, an estimate of Θ_D can be obtained considering the data fall within the $C_L = B'T^3$ curves with $B' = 0.0069$ $R.K^{-3}$ (red line) and $B'=0.0056$ $R.K^{-3}$(dashed red line), yielding $\Theta_D \approx 210 \pm 10$ K. This value lies within the typical range reported for MOFs.

S4. *Ab initio* calculations

Gd site	KD	*E* (cm^{-1})	g_x*	g_y*	g_z*
Gd1	4	0.700	0.001	0.001	13.967
	3	0.357	0.167	0.181	9.930
	2	0.133	3.593	3.797	5.502
	1	0.000	11.414	4.008	1.553
Gd2	4	1.037	0.015	0.017	13.940
	3	0.554	0.713	0.822	9.745
	2	0.247	5.588	5.323	4.708
	1	0.000	12.867	1.816	0.914
Gd3	4	0.489	0.020	0.024	13.927
	3	0.268	0.938	1.116	9.645
	2	0.128	6.076	5.506	4.428
	1	0.000	13.143	1.340	0.725

Table S1. *Ab initio* calculated components of the gyromagnetic tensor (g_z*, g_z*, g_z*) under the pseudospin representation, along with the energies of the four lowest Kramers Doublets (KDs) for the three non-equivalent Gd(III) sites in ***m*CB-Gd**. These components are expressed in the {*x*, *y*, *z*} coordinate system aligning with the magnetic anisotropy axes.

Gd site	KD	*E* (cm^{-1})	$\left\|\frac{7}{2},-\frac{7}{2}\right\rangle$	$\left\|\frac{7}{2},-\frac{5}{2}\right\rangle$	$\left\|\frac{7}{2},-\frac{3}{2}\right\rangle$	$\left\|\frac{7}{2},-\frac{1}{2}\right\rangle$	$\left\|\frac{7}{2},+\frac{1}{2}\right\rangle$	$\left\|\frac{7}{2},+\frac{3}{2}\right\rangle$	$\left\|\frac{7}{2},+\frac{5}{2}\right\rangle$	$\left\|\frac{7}{2},+\frac{7}{2}\right\rangle$
**Gd1**	**4**	0.700				1				
		0.700					1			
	**3**	0.357			1					
		0.357						1		
	**2**	0.133		1						
		0.133							1	
	**1**	0.000	1							
		0.000								1

Gd site	KD	*E* (cm^{-1})	$\left\|\frac{7}{2},-\frac{7}{2}\right\rangle$	$\left\|\frac{7}{2},-\frac{5}{2}\right\rangle$	$\left\|\frac{7}{2},-\frac{3}{2}\right\rangle$	$\left\|\frac{7}{2},-\frac{1}{2}\right\rangle$	$\left\|\frac{7}{2},+\frac{1}{2}\right\rangle$	$\left\|\frac{7}{2},+\frac{3}{2}\right\rangle$	$\left\|\frac{7}{2},+\frac{5}{2}\right\rangle$	$\left\|\frac{7}{2},+\frac{7}{2}\right\rangle$
**Gd2**	**4**	1.037	-0.0199		-0.3883		-0.9120		-0.1308	
		1.037		-0.1308		-0.91201		-0.38825		-0.0199
	**3**	0.554	-0.0619		-0.9183		0.37802		0.09959	
		0.554		0.09959		0.37802		-0.91834		-0.0619
	**2**	0.247		0.98639		-0.1591		0.04117		0.00455
		0.247	0.00455		0.04117		-0.1591		0.98639	
	**1**	0.000		-9.349E-4		0.00597		-0.06492		0.99787
		0.000	-0.9978		0.06492		-0.00597		9.349E-4	

Gd site	KD	*E* (cm^{-1})	$\left\|\frac{7}{2},-\frac{7}{2}\right\rangle$	$\left\|\frac{7}{2},-\frac{5}{2}\right\rangle$	$\left\|\frac{7}{2},-\frac{3}{2}\right\rangle$	$\left\|\frac{7}{2},-\frac{1}{2}\right\rangle$	$\left\|\frac{7}{2},+\frac{1}{2}\right\rangle$	$\left\|\frac{7}{2},+\frac{3}{2}\right\rangle$	$\left\|\frac{7}{2},+\frac{5}{2}\right\rangle$	$\left\|\frac{7}{2},+\frac{7}{2}\right\rangle$
**Gd3**	**4**	0.489		-0.15134		-0.89061		-0.42802		-0.0265
		0.489	0.0265		0.42802		0.89061		0.15134	
	**3**	0.268	0.0014	-0.13817	0.01686	-0.41035	-0.0077	0.89804	-0.00259	0.07546
		0.268	0.07546	0.00259	0.89804	0.0077	-0.41035	-0.01686	-0.13817	-0.0014
	**2**	0.128	0.0081	-0.2126	0.05905	0.0425	-0.19097	-0.0131	0.9554	-0.0018
		0.128	-0.0018	-0.9554	-0.0131	0.19097	0.0425	-0.05905	-0.2126	-0.0081
	**1**	0.000	0.99676	1E-8	-0.0799	-4E-8	0.00906	3.2E-7	-0.00174	-3.9E-6
		0.000	3.9E-6	-0.00174	-3.2E-7	0.00906	4E-8	-0.0799	-1E-8	0.99676

Table S2. (a) *Ab initio* calculated eigenstate composition of the eight lowest energy levels in the $\left|7/2, Sz\right\rangle$ basis.

S5. Calculation of Rabi frequencies

To demonstrate that a given qudit can function as a "processor" capable of performing quantum operations, it is essential to establish the condition of universality. This implies that any quantum superposition of the basis states can be reached from an initial state. To verify this, we calculated the Rabi frequencies for MW-induced transitions driven by B_1, linking all pairs of quantum states. We evaluated both the universality of each of the three isolated Gd(i) 8-level qudits and the universality of the entire ***m*CB-Gd** quMOF, which constitutes a 512-level qudit.

The energy levels, eigenstate compositions, and S_x and S_z matrices for a given applied field ($\mu_0 H$) and direction were computed using the *Phi code*, based on *ab initio*-derived anisotropy parameters (D_i, E_i) and assuming g_i = 2 for each Gd(i).

The Gd(1) case is particularly simple. For Gd(1) site, the anisotropy parameters are D = 0.057 cm^{-1} and E = 0, resulting in a diagonal S_z matrix. At an intermediate magnetic field, e.g. $\mu_0 H$ = 0.6 T (along the z-axis), the spectrum consists of eight non-equidistant levels, ordered by increasing energy:

	**(1)**	**(2)**	**(3)**	**(4)**	**(5)**	**(6)**	**(7)**	**(8)**
**E(cm^{-1}) / Eigenfunction Coefficients** $\|\boldsymbol{S},\pm \boldsymbol{M_S}\rangle$	0	0.2158	0.5465	0.9919	1.552	2.227	3.017	3.922
$\|7/2,+7/2\rangle$								1
$\|7/2,+5/2\rangle$							1	
$\|7/2,+3/2\rangle$						1		
$\|7/2,+1/2\rangle$					1			
$\|7/2,-1/2\rangle$				1				
$\|7/2,-3/2\rangle$			1					
$\|7/2,-5/2\rangle$		1						
$\|7/2,-7/2\rangle$	1							

The S_z matrix is:

***S*z**	**(1)**	**(2)**	**(3)**	**(4)**	**(5)**	**(6)**	**(7)**	**(8)**
**(8)**								-3.5
**(7)**							2.5	
**(6)**						1.5		
**(5)**					0.5			
**(4)**				-0.5				
**(3)**			-1.5					
**(2)**		-2.5						
**(1)**	-3.5							

and the S_x matrix, ordered according to the same states, is:

***S*z**	**(1)**	**(2)**	**(3)**	**(4)**	**(5)**	**(6)**	**(7)**	**(8)**
**(8)**					--	--	1.32	
**(7)**					--	1.73		1.32
**(6)**					1.94		1.73	
**(5)**				2		1.94		
**(4)**			1.94		2			
**(3)**		1.7		1.94				
**(2)**	1.3		1.7					
**(1)**		1.3						

The Rabi frequencies between each pair of eigenstates n_i,n_j can be calculated as:

$$\Omega_{i,j} = \mu_B g B_1 |\langle \mathrm{n}_i | 2S_x | \mathrm{n}_i \rangle|$$

Figure S4(left) presents the color map of Rabi frequencies, normalized by the driving field B_1 Ω_R/B_1 (MHz/mT), between each pair of adjacent levels for Gd(1). The results indicate that it is possible to connect all pairs of adjacent levels ($n_i \rightarrow n_{i+1}$) through non-zero resonant transitions, confirming the feasibility of performing universal quantum operations within the Gd(1) qudit.

The normalized Rabi frequencies Ω_R/B_1 (MHz/mT), at $\mu_0 H$ = 0.6 T (parallel to the z-axis) were also calculated for the other two Gd(i) sites (Gd(2) with anisotropy parameters D = 0.0829 cm^{-1} and E = -0.0117 cm^{-1}, and Gd(3) with D = 0.0375 cm^{-1} and E = -0.0065 cm^{-1}). The results are displayed in Figures S4(centre) and S4(right), respectively.

The universality condition is satisfied for the three Gd(i) sites, indicating that transitions between all states are accessible. However, the non-zero E parameters result in non-diagonal S_x matrices for Gd(2) and Gd(3), introducing transitions with lower frequencies between non-adjacent levels. Additionally, the Rabi frequency/B_1 color maps of Gd(1) and Gd(3) exhibit greater similarity, which is consistent with their closer D and E parameter values.

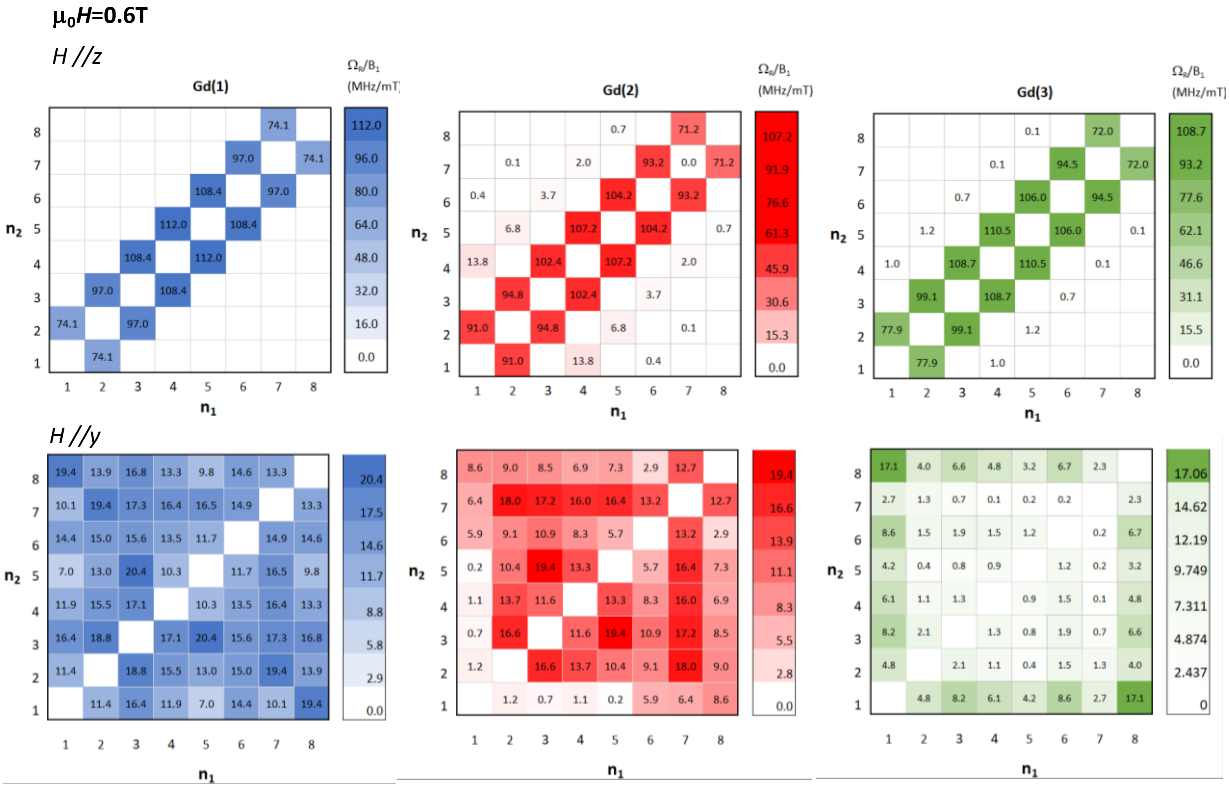

Figure S4. Rabi transition frequencies per drive field-B_1, Ω_R/B_1 (MHz/mT), between adjacent levels n_i calculated for Gd(1), Gd(2) and Gd(3) at 3.6 K under a magnetic field $\mu_0 H$ = 0.6 T applied along the z-axis (top panel) and along the y-axis (bottom panel).

It must be noted that since the three Gd(i) sites coexist within the same MOF, and their magnetic axes are not perfectly aligned, it would be impossible to orient the applied magnetic field along the *z*-axis for all Gd(i) ions simultaneously in a ***m*CB-Gd** single-crystal. Furthermore, pulsed EPR measurements to evaluate qudit performance are typically conducted on powder samples, which consist of randomly oriented grains. Hence, to address these practical considerations, it is important to analyze the normalized Rabi frequencies (Ω_R/B_1) for other magnetic field orientations. Figure S4 (bottom panel) illustrates the calculated Rabi frequencies for an applied field of 0.6 T along the *y*-axis. Results show how Rabi frequencies between adjacent levels significantly decrease along this direction, notably for Gd(3), although they do not vanish completely.

Figure S5 presents the normalized Rabi frequencies (Ω_R/B_1) calculated for the magnetic field used in our pulsed EPR experiments ($\mu_0 H$ = 0.346 T), considering field orientations along both the *z*- and *y*-directions. Additionally, Figure S6 shows the absolute Rabi frequencies (Ω_R, in MHz) calculated for a MW field B_1 = 0.54 mT at 10 dB in our EPR cavity.

Nutation experiments performed on an ***m*CB-Gd$_{0.08\%}$** powder sample at 3.6 K under $\mu_0 H$ = 0.346 T, primarily monitoring the -1/2 ↔ +1/2 transition ($n_1 = 4 \rightarrow n_2 = 5$), reveals a maximum Rabi frequency of $\Omega_R \approx 60$ MHz at 10 dB. As shown in Fig. S6, this experimental value is in good agreement with the expected Rabi frequencies for the ($n_1 = 4 \rightarrow n_2 = 5$) transition in Gd(1) and Gd(3) with *H* // z.

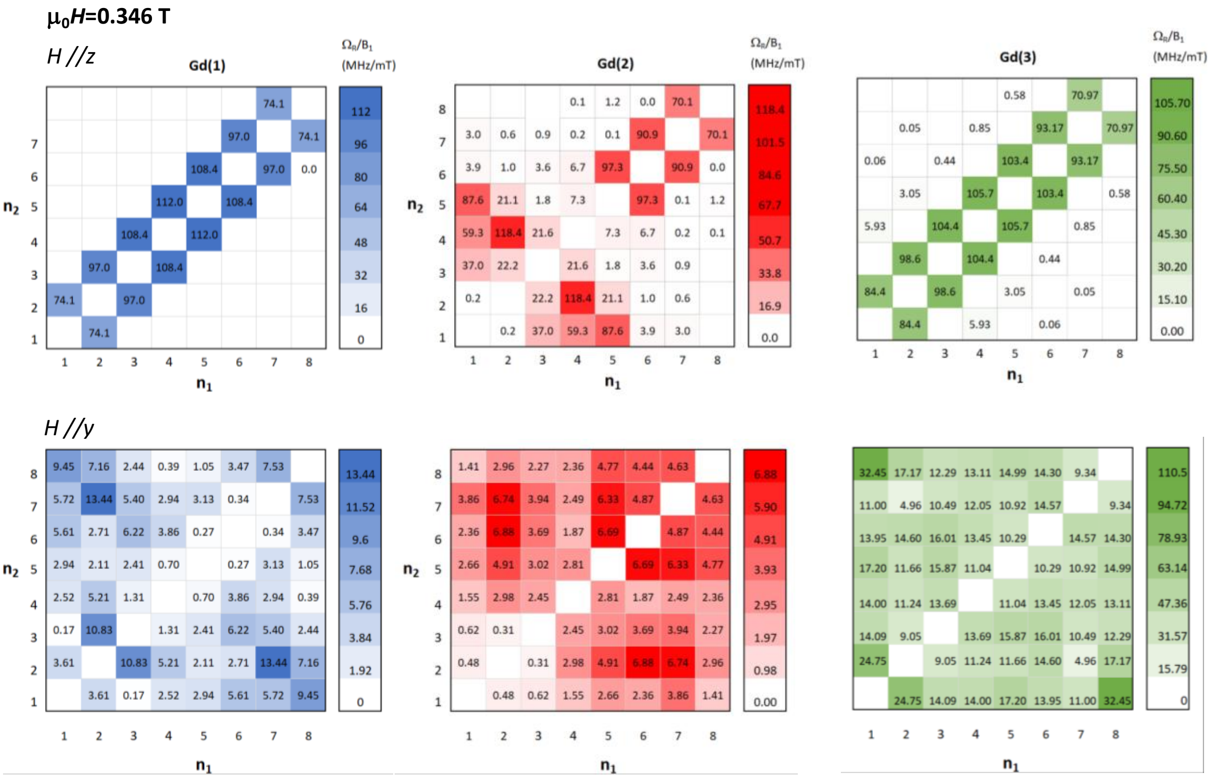

Figure S5. Normalized Rabi frequencies Ω_R/B_1 (MHz/mT) between adjacent levels calculated for Gd(1), Gd(2) and Gd(3) at 3.6 K, under a magnetic field $\mu_0 H$ = 0.346 T applied along the *z*-axis (top panel) and along the *y*-axis (bottom panel).

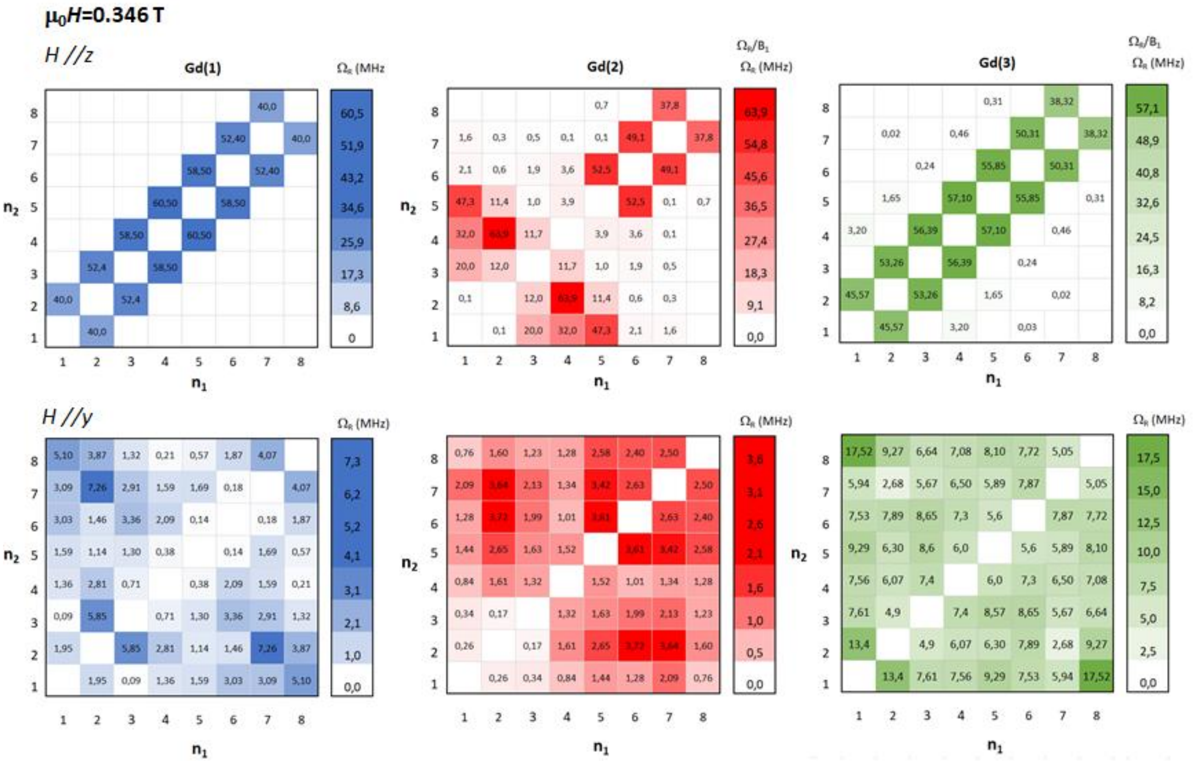

Figure S6. Rabi frequencies Ω_R(MHz) assuming B_1 = 0.45 mT (10 dB) between adjacent levels calculated for Gd(1), Gd(2) and Gd(3) at 3.6 K for a $\mu_0 H$=0.346 T magnetic field applied along the *z*-axis (top panel) and along the *y*-axis (bottom panel).

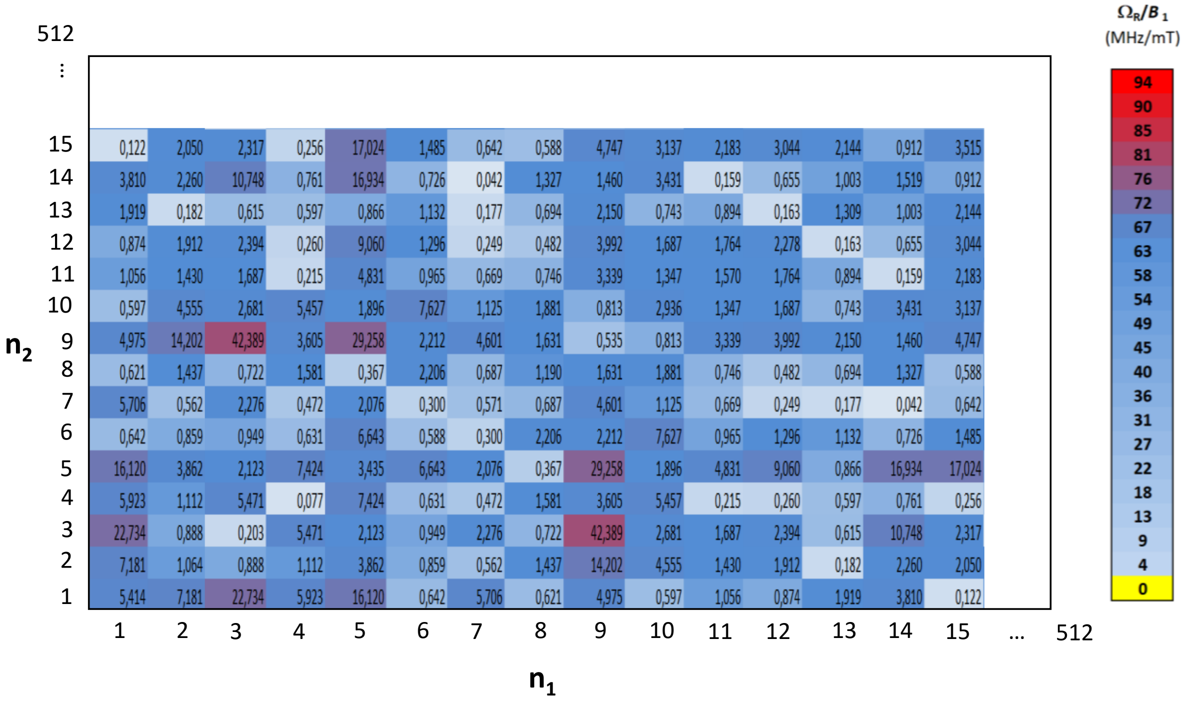

Figure S7. Color map of the (normalized) Rabi frequencies induced by B_1-driven resonant transitions between adjacent levels, calculated at 0.6 T along of the *z*-axis for ***m*CB-Gd**: zoom in of the full 512 x 512 matrix shown in Figure 6b, showing only 15 x 15 initial and final states.

S6. EPR spectroscopy

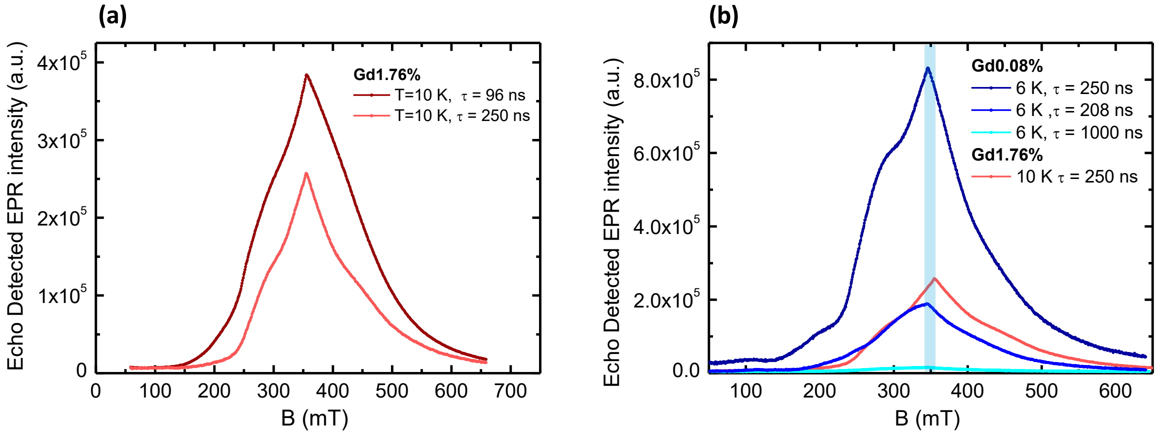

Figure S8. Field-swept echo-detected X-band EPR spectra (9.7 GHz) of magnetically diluted quMOFs: (a) ***m*CB-Gd$_{1.76\%}$** at 10 K, and (b) ***m*CB-Gd$_{0.08\%}$** at 6 K. The blue line indicates the magnetic field (346 mT), corresponding to the main (−1/2 → 1/2) transition, at which EPR nutation experiments presented in the main text were conducted. The spectra were obtained with a Hahn echo pulse sequence (π/2 - τ - π - τ -echo) under variation of the static magnetic field, using a pulse value of $t_{\pi/2}$= 16 ns, and the indicated values of τ, ranging between 96 and 1000 ns.

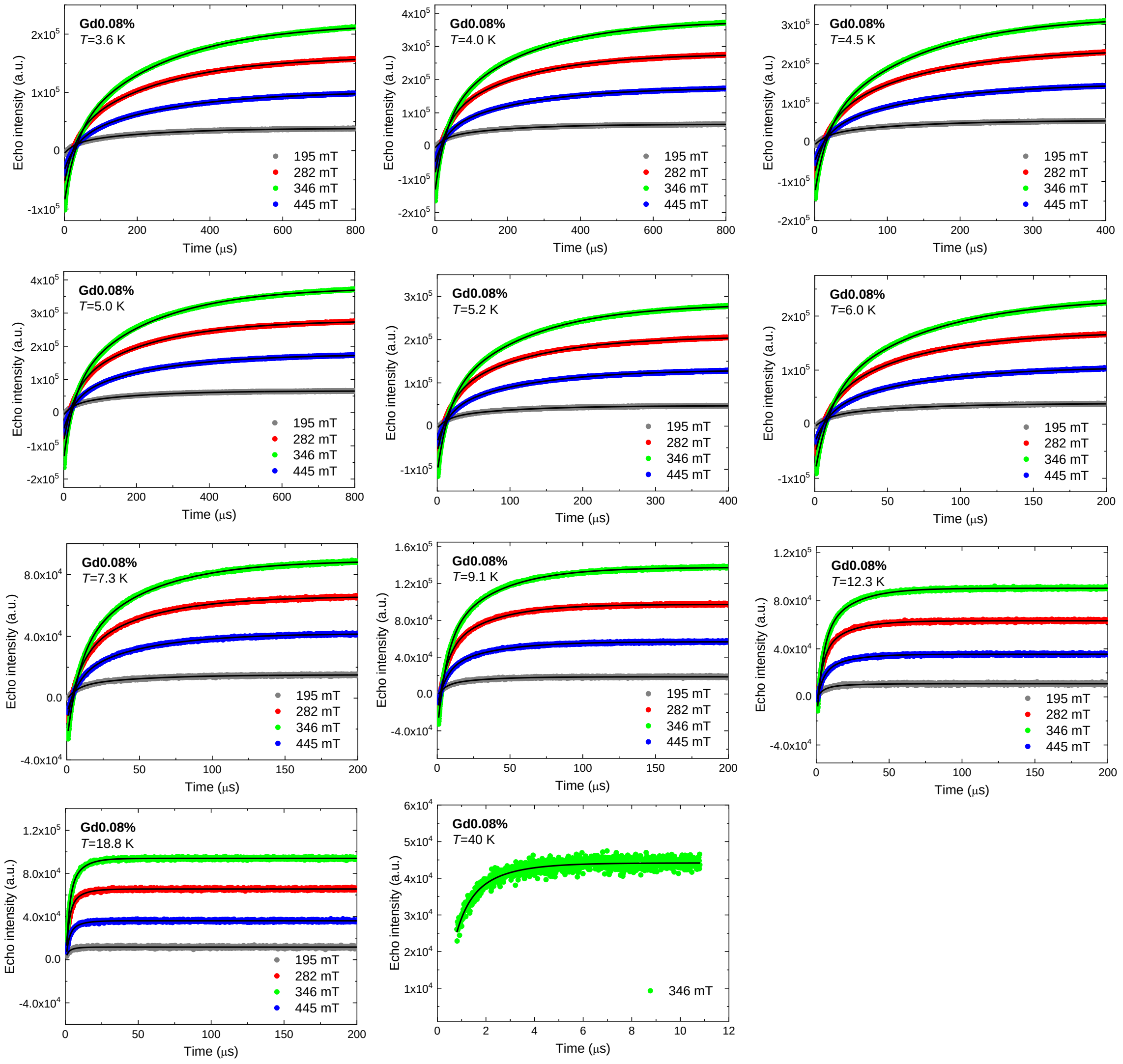

Figure S9. Echo intensity as a function of the delay time T between the inversion pulse and the detection sequence in an inversion recovery sequence (π - T - $\pi/2$ - τ - π - τ - echo) for ***m*CB-Gd$_{0.08\%}$** at different temperatures (between 3.6 K to 40 K), and magnetic field positions. The solid lines are best fits to the biexponential model given in the text, Eq. [2], with parameters given in Table S3.

Table S3. Fit parameters of T_1 to inversion recovery data for ***m*CB-Gd$_{0.08\%}$** at different temperatures between 3.6 K – 40 K, and magnetic field positions.

***T*=3.6 K**	**$T_{1,L}$ (μs)**	**$T_{1,s}$ (μs)**	***A***	***B***	**y_0**	**R^2**
**195 mT**	220.543	28.432	-25987.031	-17164.771	38587.66	0.99813
**282 mT**	245.531	31.325	-134635.992	-81533.855	161604.04	0.99951
**346 mT**	256.292	33.100	-193863.174	-112538.770	218810.58	0.99953
**445 mT**	261.892	33.641	-49635.784	-84893.547	101642.11	0.99936

***T*=4.0 K**	**$T_{1,L}$ (μs)**	**$T_{1,s}$ (μs)**	***A***	***B***	**y_0**	**R^2**
**195 mT**	25.550	186.078	-31005.671	-41046.985	65548.191	0.99836
**282 mT**	29.594	214.941	-156442.062	-207087.047	278042.293	0.99936
**346 mT**	30.805	223.159	-214785.129	-301462.126	377177.264	0.99932
**445 mT**	30.529	228.846	-94137.859	-132827.834	176103.112	0.99932

T=4.5 K	$T_{1,L}$ (μs)	$T_{1,s}$ (μs)	A	B	y_0	R^2
**195 mT**	17.038	124.097	-26987.660	-35941.509	55866.582	0.9983
**282 mT**	19.097	134.088	-129257.251	-188627.914	237348.625	0.99943
**346 mT**	17.433	133.003	-168922.695	-286140.148	321381.575	0.99967
**445 mT**	17.574	139.114	-76145.073	-124304.651	150204.543	0.99956

T=5.0 K	$T_{1,L}$ (μs)	$T_{1,s}$ (μs)	A	B	y_0	R^2
**195 mT**	25.550	186.078	-31005.671	-41046.985	65548.191	0.99836
**282 mT**	29.594	214.941	-156442.062	-207087.047	278042.293	0.99936
**346 mT**	30.805	223.159	-214785.129	-301462.126	377177.264	0.99932
**445 mT**	30.529	228.845	-94137.859	-132827.834	176103.112	0.99932

T=5.2 K	$T_{1,L}$ (μs)	$T_{1,s}$ (μs)	A	B	y_0	R^2
**195 mT**	14.417	99.058	-24437.329	-27617.280	47297.668	0.99762
**282 mT**	17.153	111.481	-122825.129	-145570.120	207489.275	0.99917
**346 mT**	16.006	112.494	-163.978	-226.267	282.667	0.99956
**445 mT**	16.487	114.745	-72661.780	-95549.930	130375.559	0.99933

T=6.0 K	$T_{1,L}$ (μs)	$T_{1,s}$ (μs)	A	B	y_0	R^2
**195 mT**	9.146	58.523	-19489.809	-23951.432	38452.876	0.99682
**282 mT**	9.001	60.091	-91996.894	-137357.112	170420.909	0.9996
**346 mT**	10.503	68.811	-136721.021	-190876.769	234383.995	0.99958
**445 mT**	9.625	65.681	-57531.206	-83747.391	106384.051	0.99942

T=7.3 K	$T_{1,L}$ (μs)	$T_{1,s}$ (μs)	A	B	y_0	R^2
**195 mT**	7.272	46.853	-8653.933	-7344.592	15106.804	0.9887
**282 mT**	8.372	46.424	-40729.616	-42016.796	65825.978	0.99922
**346 mT**	8.631	49.127	-56539.337	-61093.393	89026.866	0.99943
**445 mT**	8.113	47.963	-24487.506	-27999.219	41842.959	0.99867

T=9.1 K	$T_{1,L}$ (μs)	$T_{1,s}$ (μs)	A	B	y_0	R^2
**195 mT**	4.856	27.328	-12020.667	-9296.505	18669.848	0.97925
**282 mT**	6.007	31.997	-68181.465	-53853.277	97359.604	0.99884
**346 mT**	6.519	35.783	-98757.632	-80708.601	137531.310	0.99915
**445 mT**	6.001	33.289	-39794.660	-31017.144	56744.97856	0.99795

T=12.3 K	$T_{1,L}$ (μs)	$T_{1,s}$ (μs)	A	B	y_0	R^2
**195 mT**	4.153	18.125	-8677.212	-3707.248	10982.834	0.88491
**282 mT**	3.879	18.449	-50150.119	-27957.666	63351.964	0.99657
**346 mT**	4.272	20.364	-72861.221	-42770.889	90428.286	0.99830
**445 mT**	3.925	18.340	-27250.103	-16256.455	35510.196	0.99164

T=18.8 K	$T_{1,L}$ (μs)	$T_{1,s}$ (μs)	A	B	y_0	R^2
**195 mT**	1.327	4.772	-7598.557	-5012.798	11672.919	0.53591
**282 mT**	2.045	8.055	-55146.851	-20501.904	65511.791	0.98563
**346 mT**	2.141	8.599	-79920.614	-34691.599	93947.289	0.99348
**445 mT**	1.907	7.339	-29823.387	-12871.733	36088.9394	0.9532

T=40.0 K	$T_{1,L}$ (μs)	$T_{1,s}$ (μs)	A	B	y_0	R^2
**346 mT**	0.513	1.501	-38381.808	-18577.121	44173.424	0.90004

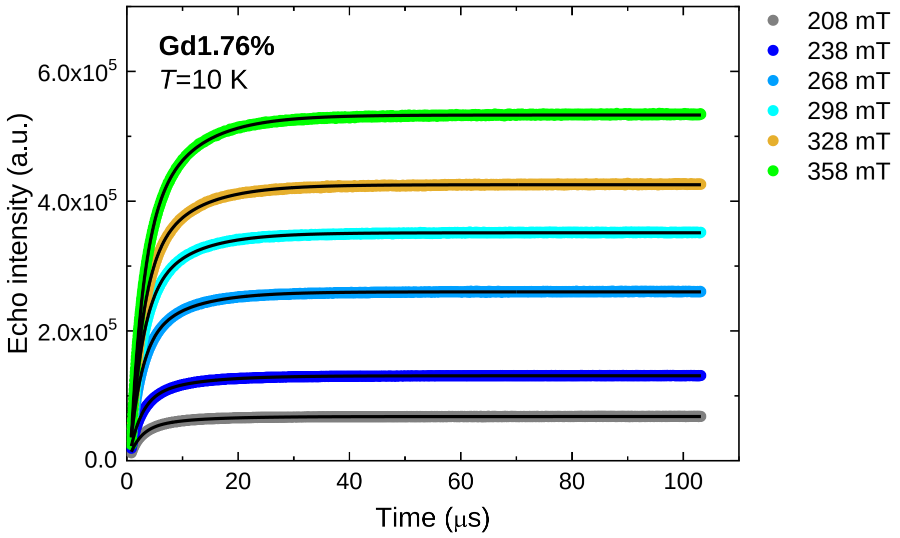

Figure S10. Echo intensity as a function of the delay time T in an inversion recovery sequence (π - t - $\pi/2$ - τ - π - τ-echo) for ***m*CB-Gd$_{1.76\%}$** at 10 K, and different field positions. The solid lines are best fits to the biexponential model described in the text, Eq. [2], with parameters given in Table S4.

Table S4. Fit parameters of T_1 to inversion recovery data for ***m*CB-Gd$_{1.76\%}$** at 10 K, and different magnetic field positions.

***T*=18.8 K**	**$T_{1,L}$ (μs)**	**$T_{1,s}$ (μs)**	***A***	***B***	**y_0**	**R^2**
**208 mT**	2.074	8.767	-53364.451	-19808.645	68076.502	0.99923
**238 mT**	2.116	8.970	-105183.712	-39914.126	130920.623	0.99938
**268 mT**	2.050	8.178	-205695.939	-94326.352	260251.454	0.99948
**298 mT**	2.179	8.276	-289760.366	-123828.697	351311.021	0.99957
**328 mT**	2.196	8.459	-358318.009	-153990.215	425366.408	0.99957
**358 mT**	2.214	8.484	-435825.253	-212601.054	532780.788	0.99959

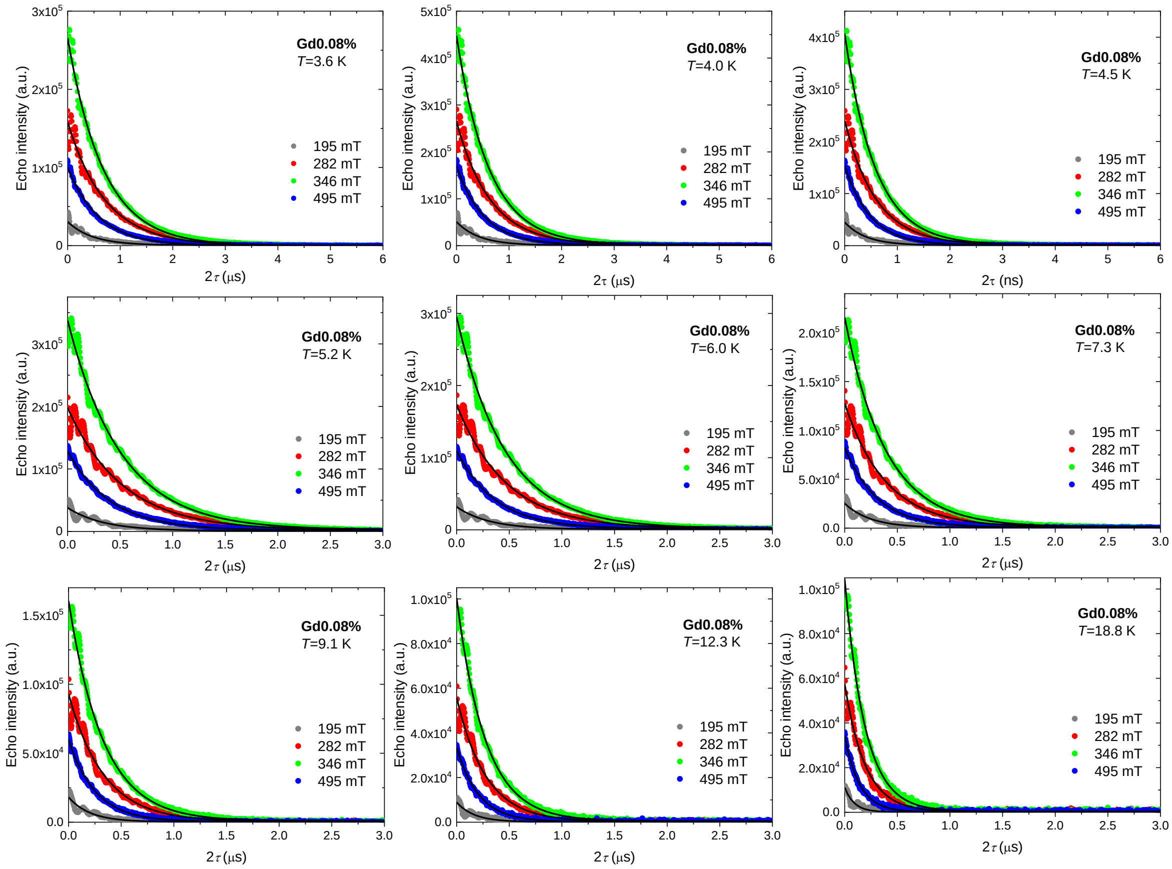

Figure S11. Echo intensity as a function of 2τ in a Hahn echo sequence (π/2 - τ - π – τ – echo) for **$Gd_{0.08\%}$** at different temperatures (between 3.6 K to 18.8 K, and magnetic field positions. The solid lines are best fits to the exponential model given in the text, Eq. [3], with parameters given in Table S5.

Table S5. Fit parameters of T_m to Hahn echo decay data for ***m*CB-$Gd_{0.08\%}$** at different temperatures between 3.6 K – 18.8 K, and magnetic field positions.

***T*=3.6 K**	**T_m (μs)**	***A***	**y_0**	**R^2**
**195 mT**	0.474	30106.321	428.051	0.94151
**282 mT**	0.716	157944.143	0	0.98989
**346 mT**	0.678	265660.001	0	0.99555
**495 mT**	0.591	100171.566	0	0.99518

***T*=4.0 K**	**T_m (μs)**	***A***	**y_0**	**R^2**
**195 mT**	0.432	50173.661	669.600	0.94112
**282 mT**	0.643	263858.199	1146.608	0.9899
**346 mT**	0.615	445980.189	1697.452	0.99574
**495 mT**	0.519	169073.722	1190.907	0.99615

***T*=4.5 K**	**T_m (μs)**	***A***	**y_0**	**R^2**
**195 mT**	0.410	44403.074	534.565	0.94457
**282 mT**	0.600	239044.748	880.9492	0.98993
**346 mT**	0.577	405450.688	1273.020	0.99601
**495 mT**	0.483	152756.055	901.665	0.99681

***T*=5.2 K**	**T_m (μs)**	***A***	***y_0***	***R^2***
**195 mT**	0.338	37188.965	822.2693	0.93595
**282 mT**	0.524	196229.199	1637.153	0.98738
**346 mT**	0.506	335169.175	2520.884	0.99495
**495 mT**	0.408	126559.517	1791.384	0.99674

***T*=6.0 K**	**T_m (μs)**	***A***	***y_0***	***R^2***
**195 mT**	0.310	31232.689	564.440	0.93472
**282 mT**	0.475	172053.735	1070.906	0.98755
**346 mT**	0.462	293131.096	1775.349	0.99491
**495 mT**	0.372	108295.967	1125.490	0.99704

***T*=7.3 K**	**T_m (μs)**	***A***	***y_0***	***R^2***
**195 mT**	0.246	24910.394	382.309	0.93458
**282 mT**	0.394	126693.463	774.637	0.98643
**346 mT**	0.389	214669.903	1145.799	0.99486
**495 mT**	0.307	82203.168	657.156	0.99637

***T*=9.1 K**	**T_m (μs)**	***A***	***y_0***	***R^2***
**195 mT**	0.205	17890.266	235.704	0.92899
**282 mT**	0.334	92482.020	427.353	0.98541
**346 mT**	0.327	159663.097	816.203	0.99453
**495 mT**	0.256	59390.298	352.460	0.99598

***T*=12.3 K**	**T_m (μs)**	***A***	***y_0***	***R^2***
**195 mT**	0.167	8791.168	136.719	0.8548
**282 mT**	0.278	55649.477	179.436	0.98535
**346 mT**	0.271	99840.339	481.125	0.99442
**495 mT**	0.209	33365.912	181.396	0.99287

***T*=18.8 K**	**T_m (μs)**	***A***	***y_0***	***R^2***
**195 mT**	0.092	10988.703	114.868	0.81412
**282 mT**	0.195	57366.306	115.578	0.9853
**346 mT**	0.192	103966.331	392.343	0.99375
**495 mT**	0.142	34057.419	129.569	0.986

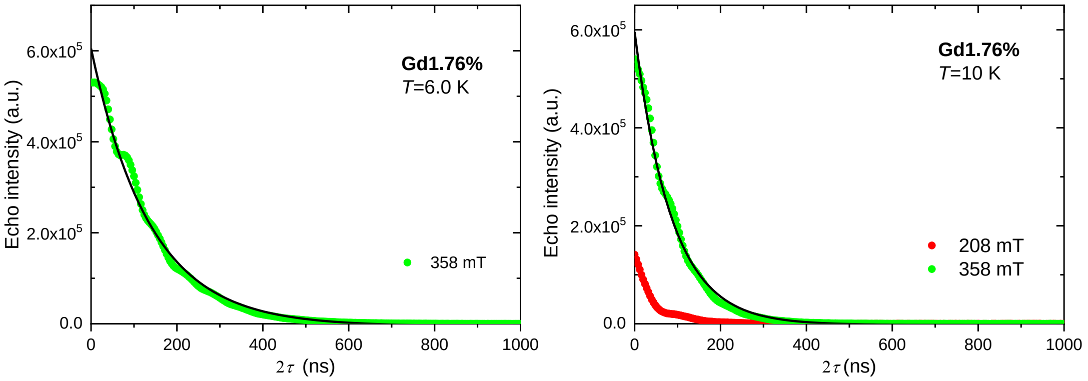

Figure S12. Echo intensity as a function of 2τ in a Hahn echo sequence (π/2 - τ - π – τ – echo) for ***m*CB-Gd$_{1.76\%}$** at different temperatures (between 3.6 K to 18.8 K, and magnetic field positions. The solid lines are best fits to the exponential model given in the text, Eq. [3], with parameters given in Table S6.

Table S6. Fit parameters of T_m to Hahn echo decay data for ***m*CB-Gd$_{1.76\%}$** at 6 K and 10 K, at 358 mT field position.

***T*=6.0 K**	**T_m (μs)**	***A***	**y_0**	**R^2**
**358 mT**	0.137	610259.341	-5268.909	0.99298

***T*=10.0 K**	**T_m (μs)**	***A***	**y_0**	**R^2**
**358 mT**	0.086	597733.340	-2226.138	0.99501

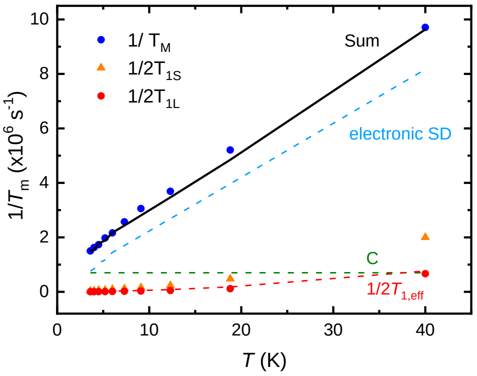

Figure S13. Inverse of the phase memory time $1/T_m$ *vs*. T data for ***m*CB-Gd$_{0.08\%}$** and fit with:

$$\frac{1}{T_m} = \frac{1}{2T_1} + A_{SD,e}\frac{1}{\sqrt{T_1}} + C.$$

In this equation, the first term accounts for decoherence due to spin–lattice relaxation, the second models the contribution from electronic spectral diffusion (SD), and the final

temperature-independent term encompasses electron spin-flop, nuclear SD, and instantaneous diffusion (ID) [1]. Since T_1 follows a biexponential decay, we used a rate-weighted effective value, $T_{1,\mathrm{eff}}$ =(A+B)/(A/ $T_{1\mathrm{S}}$+B/ $T_{1\mathrm{L}}$); C = 7 x 10^5 s, and $A_{\mathrm{SD,e}}$ = 6.6 x 10^6 $s^{-1/2}$.

References

[1] “Optimizing the spin qubit performance of lanthanide-based metal–organic frameworks”, X. Du, L. Sun, *Inorg. Chem. Front*. 2024, 11, 8660.
